# Photothermal interactions in micropolar generalized thermoelastic medium subjected to electromagnetic field

**DOI:** 10.1038/s41598-025-23882-3

**Published:** 2025-11-12

**Authors:** A. F. Al-Hazaemh, A. M. Abd-Alla, S. E. Abbas

**Affiliations:** Mathematics Department, Faculty of Science, Sohag, Egypt

**Keywords:** Thermoelasticity, Micropolar elasticity, Photothermal effect, Electro-magnetic field, Lame’s potentials, Normal mode technique, Materials science, Mathematics and computing

## Abstract

The article deals with the response of an infinite micropolar generalized thermoelastic half-space in context of Green-Naghdi theory (type III) which is subjected to electro-magnetic field. The model is used to study the coupling between elastic waves and plasma waves generated due to thermal changes in a micropolar elastic medium. The analytical expressions of displacement components, stresses components, carrier density, microrotation, couple stress and temperature are obtained by normal mode analysis technique. Effects of time, angular frequency, magnetic field, wave number, laser pulse time, and electric permittivity are highlighted on various physical fields such as displacement components, stress components, microrotation, couple stress, temperature distribution, and carrier density. The values of physical fields are computed numerically using Mathematica software considering material constants for silicon. The above physical quantities also conform to the boundary conditions. The work includes detailed graphical representations of crucial discoveries such as displacement components, temperature distributions, stress components, couple stress, microrotation and carrier density which provide amazing visual insights into the complex interactions that occur within thermo-elastic systems. The comparison of our results for the accuracy of physical quantities with previous research work is carried out graphically that indicates to the strong impact of the external parameters in photothermal phenomenon. The study is valuable for the analysis of themoelastic problems involving electro-magnetic field, micropolarity and elastic deformation.

**Supplementary Information:**

The online version contains supplementary material available at 10.1038/s41598-025-23882-3.

## Introduction

The dynamic theory of thermoelasticity has inspired many researchers to stud the thermal effect in solids. The classical theory of coupled thermoelasticity was introduced by Biot^1^ which was suffering from the parabolic nature of the heat conduction equation, anticipating infinite velocities of thermal signals, so opposing the actual physical phenomena, and therefore producing physically impossible results. So, in order to remove this disorder, several generalizations were introduced. The first generalization was established by Lord and Shulman^2^ with one relaxation time named as LS theory, and another generalization was given by Green and Lindsay^3^ with two relaxation times named as GL theory. In above LS and GL theories, the heat conduction equation is of hyperbolic nature and therefore removes the shortcoming of indefinite velocities of thermal signals. Later on, three additional models were introduced by Green and Naghdi^4–6^ (GN theory), namely G-N type I, II, and III.

Semiconducting materials serve an important role in modern engineering, and the electrical conductivity of these materials lies between conductors and insulators. One of the most important characteristics of semiconducting materials is that they possess optical properties. Therefore, when they are subjected to sun light or a laser beam or a high temperature, some of the energy will be soaked up, whereas some of the energy will be liberated in the form of heat or thermal energy. This phenomenon is known as the photothermal effect. This effect is often utilized to measure the thermal properties of the materials, especially in the semiconductor industry. In modern medicine, Photothermal technology is utilized as a noninvasive, selective treatment strategy for many different types of cancer. This technique is based on the conversion of light energy into heat on near infrared laser irradiation. This is one of the most recent applications of this technology^7^. Furthermore, when a laser beam or a sunlight or a high temperature is imposed on an elastic medium, a free charge carrier emerges on the surface, which creates the Plasma waves. In other words, plasma waves are developed due to the excited electrons, which move randomly on the surface of semiconducting material. The semiconducting material (wafer) is important to study, due to its uses in measuring temperature, thermal diffusivities, sound velocity, surface thickness, and specific heats. Different theories of thermoelasticity were proposed in the past to explore new ideas. Many articles^8–13^ have been published to discuss the coupling of temperature field with other variables like displacement etc. Nazir and Kumar^14^ investigated the photo-thermo-elastic interactions in micropolar generalized thermoelasticity theory in the framework of Green-Naghdi theory. Jatain et al.^15^ studied the thermodynamical interactions in a micropolar magneto-thermoelastic medium with photothermal effect. Mahdy et al.^16^ found the analytical solution of magneto-photothermal theory during variable thermal conductivity of a semiconductor material due to pulse heat flux and volumetric heat source. Lotfy et al.^17^ Examined the magneto-photo-thermo-microstretch semiconductor elastic medium due to photothermal transport process. Abouelregal et al.^18^ analyzed the a modified spatiotemporal nonlocal thermoelasticity theory with higher-order phase delays for a viscoelastic micropolar medium exposed to short-pulse laser excitation. Raddadi et al.^19^ explored the generalized photo-thermo-microstretch elastic solid semiconductor medium due to the excitation process. Shakeriaski et al.^20^ inspected the recent advances in generalized thermoelasticity theory and the modified models. Mahdy et al.^21^ probed the analytical solution of magneto-photothermal theory during variable thermal conductivity of a semiconductor material due to pulse heat flux and volumetric heat source. Raddadi et al.^22^ researched the novel generalized photo-thermoelasticity model for hydroporoelastic semiconductor medium. Hilal et al.^23^ scrutinized the phase-lag and diffusion in porous thermoelastic micropolar media with initial pressure and rotational forces affected by modified Ohm’s law and gravity. Raddadi et al.^24^ checked the rotating magneto-photo-thermoelastic effect with moisture diffusivity of nonlocal semiconductor medium. Ailawalia and Sachdeva^25^ investigated the internal heat source in a temperature dependent thermoelastic half space with micro-temperatures. Saeed et al.^26^ Inquired the hall current effect of magnetic-optical-elastic-thermal-diffusive semiconductor model during electrons-holes excitation processes. Ailawalia et al.^27^ verified the behavior of functionally graded semiconducting rod with internal heat source under a thermal shock. Photothermal effect is associated with electromagnetic radiation. It is produced by the photoexcitation of material, resulting in the production of thermal energy. In modern engineering, photothermal effect is used in semiconductors, integrated chips, etc. Salah et al.^28^ investigated the effect of magnetic field and initial stress on a rotating photothermal semiconductor medium with ramp type heating and internal heat source. In recent years, researchers have extensively focused on the wave’s propagation in a photothermal semiconducting medium (see for example^28–40^ and several references therein. Photothermal effect is associated with electromagnetic radiation. It is produced by the photoexcitation of material, resulting in the production of thermal energy. In modern engineering, photothermal effect is used in semiconductors, integrated chips, etc. Song et al.^37^ studied the reflection of photothermal waves under generalized thermoelastic theory and obtained the analytical expressions for the reflection coefficients.

The present paper delves into interaction between plasma waves and elastic waves generated due to the thermal changes induced in the micropolar solid in the presence of electro-magnetic field. The article also tends to study the effect of some parameters on the various field quantities (displacement, stress, couple stress, temperature, carrier density, etc.) for the given micropolar solid. The numerical results are obtained for silicon-like material, and graphs have been drawn to highlight the effects. The obtained results are then compared with previous studies in the same field, revealing the effectiveness of the chosen approach in solving the given problem. Given the realistic composition of this type of model within the Earth’s interior, its applications extend to critical structures such as aerospace materials, nuclear reactors, pressure vessels, and pipelines. Ultimately, the findings of this research are evaluated in relation to other published studies to highlight their significance.

## Basic equations

The field equations of motion and constitutive relations for the micropolar theory of generalized thermoelasticity in the context of photothermal theory is given by:

(i) The equations of motion for a thermoelastic semiconductor material are:
  1 $$(\lambda + \mu )\underline{\nabla } (\underline{\nabla } \cdot \overrightarrow {u} ) + (\mu + k)\nabla^{2} \overrightarrow {u} + k(\underline{\nabla }^{ \wedge } \overrightarrow {u} - \beta_{1} \underline{\nabla } \theta - \gamma_{n} \underline{\nabla } N = \rho \frac{{\partial \overrightarrow {u} }}{{\partial t^{2} }},$$
(ii) The equations of micropolar material are
  2 $$(\alpha + \beta + \gamma )\underline{\nabla } (\underline{\nabla } .\,\,\overrightarrow {\varphi } ) - \gamma \underline{\nabla }^{ \wedge } (\underline{\nabla }^{ \wedge } \overrightarrow {\varphi } ) + k(\underline{\nabla }^{ \wedge } \overrightarrow {\varphi )} - 2k\overrightarrow {\varphi } = \rho j\frac{{\partial \overrightarrow {\varphi } }}{{\partial t^{2} }},$$
(iii) The stress introduces from body force take the form
  3 $$\sigma_{ij} = (\lambda u_{r,r} - \beta_{1} \theta - \gamma_{n} N)\delta_{ij} + \mu (u_{i,j} + u_{j,i} ) + k(u_{j,i} - \varepsilon_{ijr} \varphi_{r} ),$$
(iv) The couple stress
  4 $$M_{ij} = \alpha \varphi_{r,r} \delta_{ij} + \beta \varphi_{i,j} + \gamma \varphi_{j,i} .\,\,$$
(v) Following Nazir and Kumar^14^, the heat conduction equation for a micropolar elastic semiconductor sample can be written as:
  5 $$\left( {K^{*} + K\frac{\partial }{\partial t}} \right)\nabla^{2} \theta + \frac{{E_{g} }}{\tau }N - \rho c_{e} \frac{{\partial^{2} \theta }}{{\partial t^{2} }} - \gamma_{t} T_{0} \frac{{\partial^{2} e}}{{\partial t^{2} }} = 0.$$
(vi) According to Nazir and Kumar^14^, the coupled plasma and thermal distribution equation can be written as:
  6 $$D_{e} \,\nabla^{2} N - \frac{\partial N}{{\partial t}} - \frac{N}{\tau } + \frac{\delta \theta }{\tau } = 0..$$

## Formulation of the problem

We consider a homogeneous, isotropic, micropolar, electro-magneto-thermoelastic semiconducting medium with photothermal effect. This study is taken into consideration under the Green-Naghdi theory (type III). Also, the origin of a rectangular Cartesian coordinate system $\left( {x,y,z} \right)$ is taken at any point on the plane surface of a half-space, $x = 0$ for the 2D problem, the displacement vector $\overrightarrow {u} = (u,\,0,\,w)$ and microrotation vector $\overrightarrow {\varphi }$ is given by $\overrightarrow {\phi } = \left( {0,\phi_{2} ,0} \right).$ Let us consider that there are Maxwell equations (governing the electromagnetic field) with the assumption that the elastic solid (Epoxy resin) medium is perfectly electric conductor are given by Xiong and Tian^29^.

The Maxwell equations of the electromagnetic field can be found as follows when the electro-dynamic medium is linearized for slow motion:

$$\overrightarrow {j} = curl\overrightarrow {h} - \varepsilon_{0} \frac{{\partial \overrightarrow {E} }}{\partial t},\,\,\,\, - \mu_{e} \frac{{\partial \overrightarrow {h} }}{\partial t} = curl\overrightarrow {E,} \,\,\,\,\,div\overrightarrow {h} = o,\,\,\,\,div\overrightarrow {E} = 0,$$

7 $$\vec{E} = - \mu_{e} \left( {\frac{{\partial \vec{u}}}{\partial t} \times \vec{H}} \right), \,\,\,\,\vec{h} = curl \left( {\vec{u} \times \vec{H}} \right)$$

where, $\varepsilon_{0}$ and $\mu_{e}$ represent the electric permittivity and magnetic permeability, respectively. Also, ($\overrightarrow {{\dot{u}}}$) denotes the particle velocity ($\overrightarrow {u}$ is the displacement vector), and the dot notation denotes the time differentiation, the vector $\vec{J}$ represents the current density in the semiconductor medium, which may be taken in the direction of the electric field $\vec{E}$, the magnetic field intensity vector and the electric field intensity vector are denoted by $\vec{h}(H = H_{0} + h)$ and $\vec{E}$ respectively is expressed as follow:

8 $$\begin{aligned} & H_{x} = H_{z} = 0,\,\,H_{y} = H_{0} \\ & \vec{E} = - \mu_{e} \left| {\begin{array}{*{20}c} {\vec{i}} & {\vec{J}} & {\vec{k}} \\ {\dot{u}} & 0 & {\dot{w}} \\ 0 & {H_{0} } & 0 \\ \end{array} } \right| \\ & E_{x} = \mu_{e} H_{0} \dot{w}, \,\,E_{y} = 0,\,\,E_{z} = - \mu_{e} H_{0} \dot{u} \\ \end{aligned}$$

From Eq. (7), we can calculate the vector components of the current density in terms of the displacement component as

9 $$\begin{aligned} & \vec{J} = \left| {\begin{array}{*{20}c} {\vec{i}} & {\vec{J}} & {\vec{k}} \\ {\frac{\partial }{\partial x}} & {\frac{\partial }{\partial y}} & {\frac{\partial }{\partial z}} \\ 0 & {H_{0} } & 0 \\ \end{array} } \right| - \varepsilon_{^\circ } \left( {0, 0, - \mu_{e} H_{0} \dot{u} } \right) \\ & J_{x} = J_{y} = 0, \,\,\,\,J_{z} = \frac{\partial h}{{\partial x}} + \mu_{e} H_{0} \varepsilon_{0} \dot{u} \\ \end{aligned}$$

The Lorentz force vector takes the following form

10 $$\overrightarrow {F} = \mu_{e} (\overrightarrow {J} x\overrightarrow {H} ).$$

where

$$\overrightarrow {H} = (0,H_{0} ,0),\,\,\,\,\,\,\,\,F_{x} = \frac{{\partial \tau_{xx} }}{\partial x},\,\,\,\,\,\,\,\,F_{z} = \frac{{\partial \tau_{zz} }}{\partial z},$$

$F_{x}$ and $F_{z}$ are the components of Lorentz forces.

The dynamic equations of motion (1) subjected to electro-magnetic field, are given by:

11 $$\begin{aligned} & \left( {\lambda + \mu } \right)\left( {\frac{{\partial^{2} u}}{{\partial x^{2} }} + \frac{{\partial^{2} w}}{\partial x\partial z}} \right) + \left( {\mu + k} \right)\nabla^{2} u - k\frac{{\partial \varphi_{2} }}{\partial z} - \beta_{1} \frac{\partial \theta }{{\partial x}} - \gamma_{n} \frac{\partial N}{{\partial x}} \\ & \,\,\,\, + H_{0 }^{2} \mu_{e } \left( {\frac{{\partial^{2} u}}{{\partial x^{2} }} + \frac{{\partial^{2} w}}{\partial x\partial z}} \right) - - \epsilon_{0} \mu_{e}^{2} H_{0 }^{2} \frac{{\partial^{2} u}}{{\partial t^{2} }} = \rho \frac{{\partial^{2} u}}{{\partial t^{2} }} \\ \end{aligned}$$

12 $$\begin{aligned} & \left( {\lambda + \mu } \right)\left( {\frac{{\partial^{2} u}}{\partial x\partial z} + \frac{{\partial^{2} w}}{{\partial z^{2} }}} \right) + \left( {\mu + k} \right)\nabla^{2} w + k\frac{{\partial \varphi_{2} }}{\partial x} - \beta_{1} \frac{\partial \theta }{{\partial z}} - \gamma_{n} \frac{\partial N}{{\partial z}} \\ & \,\,\,\, + H_{0 }^{2} \mu_{e } \left( {\frac{{\partial^{2} u}}{\partial x\partial z} + \frac{{\partial^{2} w}}{{\partial z^{2} }}} \right) - \epsilon_{0} \mu_{e}^{2} H_{0 }^{2} \frac{{\partial^{2} w}}{{\partial t^{2} }} = \rho \frac{{\partial^{2} w}}{{\partial t^{2} }} \\ \end{aligned}$$

The micropolar theory of thermoelasticity in the context of photothermal theory (2) is given by:

13 $$\gamma \nabla^{2} \varphi_{2} + k\left( {\frac{\partial u}{{\partial z}} - \frac{\partial w}{{\partial x}}} \right) - 2k\varphi_{2} = \rho j \frac{{\partial^{2} \varphi_{2} }}{{\partial t^{2} }}$$

The coupling between Plasma and thermoelastic waves as defined in Raddadi et al.^19^, can be written as:

14 $$D_{e} \nabla^{2} N - \frac{\partial N}{{\partial t}} - \frac{N}{\tau } + \frac{\delta \theta }{\tau } = 0$$

where $D_{e}$ is the coefficient of carrier diffusion, N = n − $n_{0}$, $n_{0}$ represents equilibrium carrier concentration , τ is the photogenerated carrier lifetime, δ = $\frac{{\partial n_{0} }}{\partial \theta }$ is the coupling parameter of thermal activation.

The heat conduction equation in the presence of photothermal theory under Green-Naghdi theory (type III) as defined in^27^, is given by:

15 $$\left( {K^{*} + k\frac{\partial }{\partial t}} \right)\nabla^{2} \theta = \frac{{E_{g} }}{\tau } N + \frac{{\partial^{2} }}{{\partial t^{2} }}\left( {\rho c_{e} \theta + \gamma_{t} T_{0} \left( {\frac{\partial u}{{\partial x}} + \frac{\partial w}{{\partial z}}} \right)} \right).$$

The constitutive stress and couple stress relations are expressed as, (Jatain et al.^15^):

$$\sigma_{xx} = \left( {\lambda + 2\mu + k} \right)\frac{\partial u}{{\partial x}} + \lambda \frac{\partial w}{{\partial z}} - \beta_{1} \theta - \gamma_{n} N,$$

$$\sigma_{zz} = \left( {\lambda + 2\mu + k} \right)\frac{\partial w}{{\partial z}} + \lambda \frac{\partial u}{{\partial x}} - \beta_{1} \theta - \gamma_{n} N,$$

$$\sigma_{xz} = \mu \frac{\partial u}{{\partial z}} + \left( {\mu + k} \right)\frac{\partial w}{{\partial x}} + k\varphi_{2} ,$$

$$\sigma_{zx} = \mu \frac{\partial w}{{\partial x}} + \left( {\mu + k} \right)\frac{\partial u}{{\partial z}} - k\varphi_{2} ,$$

16 $$M_{zy} = \gamma \frac{{\partial \varphi_{2} }}{\partial z} ,$$

$$M_{xy} = \gamma \frac{{\partial \varphi_{2} }}{\partial x}.$$

In the above Eqs. (11–16), $\beta_{1}$ = (3λ + 2μ + κ)$\alpha_{t}$, $\gamma_{n}$ = (3λ + 2μ)$d_{n}$, where $d_{n}$ represents electronic deformation coefficient.

## Boundary conditions

The plane surface $x = 0$ of the micropolar electro-magneto-thermoelastic medium with photothermal effect is subjected to an arbitrary thermal load. Therefore, the boundary conditions are given by:

The boundary conditions of the problem on $x=0$, can be expressed as:

1. Mechanical boundary conditions at the surface *x* = 0

17 $$\begin{gathered} \sigma_{xx} = 0,\,\,\,\,\, \hfill \\ \,\sigma_{xz} = 0,\,\,\,\,\, \hfill \\ \,M_{xy} = 0. \hfill \\ \end{gathered}$$

2. When x = 0, the thermally gradient temperature can be used to depict the pulsating heat flow boundary condition in the following ways:

18 $$\frac{\partial \theta }{{\partial x}} = - q_{0} \frac{{t^{2} e^{{ - \frac{t}{{t_{p} }}}} }}{{16kt_{p}^{2} }},$$

3. Boundary condition for diffusion process at the surface $x = 0:$

The carriers arrive at the surface with the possibility of limited recombination having recombination velocity s. This gives:

19 $$\frac{\partial N}{{\partial x}} - \frac{{s_{0} }}{{D_{e} }}N = 0.$$

where $t_{p}$ is the characteristic time of pulse heat flux, $s_{0}$ is the surface recombination speed and $q_{0}$ is a constant.

Now, the non-dimensional quantities are defined as follows

20 $$\begin{gathered} \left( {x^{\prime},z^{\prime}} \right) = \frac{{\omega^{*} }}{{c_{1} }}\left( {x,z} \right),\left( {u^{\prime},w^{\prime}} \right) = \frac{{\rho \omega^{*} c_{1} }}{{T_{0} \beta_{1} }}\left( {u,w} \right),\theta^{\prime} = \frac{\theta }{{T_{0} }},\sigma_{ij}{\prime} = \frac{{\sigma_{ij} }}{{T_{0} \beta_{1} }},\varphi^{\prime}_{2} \hfill \\ = \frac{{\rho c_{1}^{2} }}{{T_{0} \beta_{1} }}\varphi_{2} ,M_{ij}{\prime} = \frac{{\omega^{*} }}{{c_{1} T_{0} \beta_{1} }}M_{ij} , \left( {t^{\prime},\tau^{\prime},t_{p}{\prime} } \right) = \omega^{*} \left( {t,\tau ,t_{p} } \right), N^{\prime} = \frac{N}{{n_{0} }} , q_{0}{\prime} = \frac{{q_{0} c_{1} }}{{\omega^{*} T_{0} K}} \hfill \\ {\text{where}}, \omega^{*} = \frac{{\rho c_{1}^{2} c_{e} }}{{K^{*} }} , c_{1}^{2} = \frac{{\left( {\lambda + 2\mu + k} \right)}}{\rho } \hfill \\ \end{gathered}$$

After using the non-dimensional parameters defined in (20), Eqs. (11–16) become (dropping the prime notation):

21 $$\nabla^{2} u + \propto_{1} \left( {\frac{{\partial^{2} u}}{{\partial x^{2} }} + \frac{{\partial^{2} w}}{\partial x\partial z}} \right) - \propto_{2} \frac{{\partial \varphi_{2} }}{\partial z} - \propto_{3} \frac{\partial \theta }{{\partial x}} - \propto_{4} \frac{\partial N}{{\partial x}} = \propto_{6} \frac{{\partial^{2} u}}{{\partial t^{2} }},$$

22 $$\nabla^{2} w + \propto_{1} \left( {\frac{{\partial^{2} u}}{\partial x\partial z} + \frac{{\partial^{2} w}}{{\partial z^{2} }}} \right) + \propto_{2} \frac{{\partial \varphi_{2} }}{\partial x} - \propto_{3} \frac{\partial \theta }{{\partial z}} - \propto_{4} \frac{\partial N}{{\partial z}} = \propto_{6} \frac{{\partial^{2} w}}{{\partial t^{2} }},$$

23 $$\nabla^{2} \varphi_{2} + \propto_{7} \left( {\frac{\partial u}{{\partial z}} - \frac{\partial w}{{\partial x}} - 2\varphi_{2} } \right) = \propto_{8} \frac{{\partial^{2} \varphi_{2} }}{{\partial t^{2} }},$$

24 $$\nabla^{2} N = \alpha_{9} \left( {\frac{\partial N}{{\partial x}} - \frac{N}{\tau }} \right) - \alpha_{10} \frac{\theta }{\tau },$$

25 $$\left( { \propto_{17} + \frac{\partial }{\partial t}} \right)\nabla^{2} \theta = \propto_{18} \frac{{\partial^{2} \theta }}{{\partial t^{2} }} + \alpha_{19} \frac{{\partial^{2} }}{{\partial t^{2} }}\left( {\frac{\partial u}{{\partial x}} - \frac{\partial w}{{\partial z}}} \right) - \propto_{20} \frac{N}{\tau },$$

26 $$\sigma_{xx} = \frac{\partial u}{{\partial x}} + \propto_{11} \frac{\partial w}{{\partial z}} - \theta - \propto_{12} N,$$

27 $$\sigma_{zz} = \frac{\partial w}{{\partial z}} + \propto_{11} \frac{\partial u}{{\partial x}} - \theta - \propto_{12} N,$$

28 $$\sigma_{xz} = \propto_{13} \frac{\partial u}{{\partial z}} + \propto_{14} \frac{\partial w}{{\partial x}} + \propto_{15} , \varphi_{2}$$

29 $$\sigma_{zx} = \propto_{13} \frac{\partial w}{{\partial x}} + \propto_{14} \frac{\partial u}{{\partial z}} - \propto_{15} \varphi_{2} ,$$

30 $$M_{zy} = \propto_{16} \frac{{\partial \varphi_{2} }}{\partial z},$$

31 $$M_{xy} = \propto_{16} \frac{{\partial \varphi_{2} }}{\partial x}$$

32 $$\begin{gathered} where,\,\, \propto_{1} = \frac{{\left( {\lambda + \mu } \right) + H_{0 }^{2} \mu_{e } }}{{\left( {\mu + k} \right)}},\, \propto_{2} \frac{k}{{\left( {\mu + k} \right)}},\, \propto_{3} = \frac{{\rho c_{1}^{2} }}{{\left( {\mu + k} \right)}}, \propto_{4} = \frac{{\gamma_{n} n_{0 } \rho c_{1}^{2} }}{{T_{0} \beta_{1} \left( {\mu + k} \right)}}, \propto_{6} = \frac{{c_{1}^{2} }}{{\left( {\mu + k} \right)}}\left( {\rho + \epsilon_{0} \mu_{e}^{2} H_{0 }^{2} } \right), \propto_{7} = \frac{{k c_{1}^{2} }}{{\gamma \omega^{*2} }}, \hfill \\ \alpha_{8} = \frac{{\rho j c_{1}^{2} }}{\gamma }, \propto_{9} = \frac{{c_{1}^{2} }}{{D_{e} \omega^{*} }} , \alpha_{10} = \frac{{\delta T_{0} c_{1}^{2} }}{{D_{e} n_{0} \omega^{*} }} , \alpha_{11} = \frac{\lambda }{{\rho c_{1}^{2} }} ,\alpha_{12} = \frac{{\gamma_{n} n_{0} }}{{T_{0} \beta_{1} }} , \alpha_{13} = \frac{\mu }{{\rho c_{1}^{2} }} , \alpha_{14} = \frac{\mu + k}{{\rho c_{1}^{2} }} , \hfill \\ \alpha_{15} = \frac{k}{{\rho c_{1}^{2} }} , \alpha_{16} = \frac{{\gamma \omega^{*2} }}{{\rho c_{1}^{4} }} , \alpha_{17} = \frac{{k^{*} }}{{k \omega^{*} }} , \alpha_{18} = \omega^{*2} , \alpha_{19} = \frac{{\gamma + T_{0 } \beta_{1} }}{{k\rho c_{1} \omega^{*2} }} , \alpha_{20} = \frac{{E_{g} n_{0} c_{1}^{2} }}{{T_{0} k\omega^{*2} }} \hfill \\ \end{gathered}$$

## Solution of the problem

Using Helmholtz’s representation, the displacement components^31^ may be expressed.

in terms of scalar and vector potential functions

$$u = \frac{\partial \phi }{{\partial x}} - \frac{\partial \psi }{{\partial z}},$$

33 $$w = \frac{\partial \phi }{{\partial z}} + \frac{\partial \psi }{{\partial x}}.$$

Using the representations (33) in (21–23) and (25–29), we obtain the equations in decoupled form:

34 $$\frac{{\partial^{2} \phi }}{{\partial x^{2} }} + \frac{{\partial^{2} \phi }}{{\partial z^{2} }} - a_{1} \theta - a_{2 } N = a_{3 } \frac{{\partial^{2} \phi }}{{\partial t^{2} }},$$

35 $$\frac{{\partial^{2} \psi }}{{\partial x^{2} }} + \frac{{\partial^{2} \psi }}{{\partial z^{2} }} + \propto_{2} \varphi_{2} = \propto_{6} \frac{{\partial^{2} \psi }}{{\partial x^{2} }} ,$$

36 $$\nabla^{2} \varphi_{2} - \propto_{7} \left( {\frac{{\partial^{2} \psi }}{{\partial z^{2} }} + \frac{{\partial^{2} \psi }}{{\partial x^{2} }} + 2\varphi_{2} } \right) = \propto_{8} \frac{{\partial^{2} \varphi_{2} }}{{\partial t^{2} }},$$

37 $$\nabla^{2} N = \alpha_{9} \left( {\frac{\partial N}{{\partial x}} - \frac{N}{\tau }} \right) - \alpha_{10} \frac{\theta }{\tau },$$

38 $$\left( { \propto_{17} + \frac{\partial }{\partial t}} \right)\nabla^{2} \theta = \propto_{18} \frac{{\partial^{2} \theta }}{{\partial t^{2} }} - \alpha_{19} \frac{{\partial^{2} }}{{\partial t^{2} }}\left( {\frac{{\partial^{2} \psi }}{{\partial z^{2} }} + \frac{{\partial^{2} \psi }}{{\partial x^{2} }}} \right) - \propto_{20} \frac{N}{\tau }.$$

$${\text{where}} \,\,a_{1} = \frac{{ \propto_{3} }}{{1 + \propto_{1} }} , \,\,a_{2} = \frac{{ \propto_{4} }}{{1 + \propto_{1} }} , \,\,a_{3} = \frac{{ \propto_{6} }}{{1 + \propto_{1} }}$$

In this section, normal mode analysis (NMA) is used to obtain the analytical expressions for displacement components, stress components, temperature, microrotation, coupled stress and carrier density. The assumed solution of physical variables is decomposed into normal modes to obtain the analytical solutions of the physical quantities. It is assumed that all the functions are sufficiently smooth such that the NMA of these functions exist. The variables can be decomposed in terms of normal modes in the following form:

39 $$\left( {\varphi ,\psi ,\theta ,N,\varphi_{2} } \right)\left( {x,z,t} \right) = \left( {\varphi^{*} (x),\psi^{*} ,\theta^{*} ,N^{*} ,\varphi_{2}^{*} } \right)\left( x \right)e^{{\left( {\omega t + ibz} \right)}}$$

where ω is the frequency of waves propagating in the medium and $b$ is the wave number.

Using (39) in Eqs. (34–38), we obtain the following form for the equations:

40 $$\left( {D^{2} - \delta_{11} } \right)\varphi^{*} (x) - \propto_{1} \theta^{*} \left( x \right) - a_{2} N^{*} \left( x \right) = 0,$$

41 $$\left( {D^{2} - \delta_{14} } \right)N^{*} \left( x \right) + \frac{{\alpha_{10} }}{\tau }\theta^{*} \left( x \right) = 0,$$

42 $$\left( {\delta_{18} D^{2} - \delta_{13} } \right)\theta^{*} \left( x \right) + \left( {\delta_{15} - \delta_{19} D^{2} } \right)\varphi^{*} (x) = 0,$$

43 $$\left( {D^{2} - \delta_{16} } \right)\varphi_{2}^{*} (x) - \left( { \propto_{7} D^{2} + \propto_{7} b^{2} } \right)\psi^{*} \left( x \right) = 0,$$

44 $$\left( {\delta_{12} D^{2} - b^{2} } \right)\psi^{*} \left( x \right) + \alpha_{2} \varphi_{2}^{*} (x) = 0.$$

where, $${{\delta }_{11}={b}^{2}+{a}_{3 }{\omega }^{2}, {\delta }_{12}=1-{\alpha }_{6}, {\delta }_{13}={\alpha }_{17}\omega {b}^{2}+{\alpha }_{18}{\omega }^{2}, {\delta }_{14}={b}^{2}+{\alpha }_{9}\omega +\frac{{\alpha }_{9}}{\tau }, {\delta }_{15}={\omega }^{2}{\alpha }_{19}{b}^{2}, \delta }_{16}={b}^{2}+{\propto }_{8}{\omega }^{2}, {\delta }_{18}={\alpha }_{17}\omega , {\delta }_{19}={\alpha }_{19}{\omega }^{2}$$

$$\begin{aligned} \beta _{{11}} = & \propto _{{17}} \omega ,\beta _{{22}} = 3b^{2} \propto _{{17}} \omega + \frac{{ \propto _{{17}} \propto _{9} \omega }}{\tau } + \propto _{{18}} \omega ^{2} + \propto _{1} \propto _{{19}} \omega ^{2} + \propto _{{17}} \propto _{9} \omega ^{2} + \propto _{3} \propto _{{17}} \omega ^{3} , \\ \beta _{{33}} = & 3b^{4} \propto _{{17}} \omega + \frac{{2b^{2} \propto _{{17}} \propto _{9} \omega }}{\tau } + 2b^{2} \propto _{{18}} \omega ^{2} + 2b^{2} \propto _{1} \propto _{{19}} \omega ^{2} + 2b^{2} \propto _{{17}} \propto _{9} \omega ^{2} \\ & + \frac{{ \propto _{2} \propto _{{10}} \propto _{{19}} \omega ^{2} }}{\tau } + \frac{{ \propto _{{18}} \propto _{9} \omega ^{2} }}{\tau } + \frac{{ \propto _{1} \propto _{{19}} \propto _{9} \omega ^{2} }}{\tau } + 2a_{3} b^{2} \propto _{{17}} \omega ^{3} + \propto _{{18}} \propto _{9} \omega ^{3} + \propto _{1} \propto _{{19}} \propto _{9} \omega ^{3} \\ & + \frac{{ \propto _{3} \propto _{{17}} \propto _{9} \omega ^{3} }}{\tau } + \propto _{3} \propto _{{18}} \omega ^{4} + a_{3} \propto _{{17}} \propto _{9} \omega ^{4} , \\ \beta _{{44}} = & - b^{6} \propto _{{17}} \omega - b^{4} \propto _{{18}} \omega ^{2} - b^{4} \propto _{1} \propto _{{19}} \omega ^{2} - b^{4} \propto _{{17}} \propto _{9} \omega ^{2} - \frac{{a_{2} b^{2} \propto _{{10}} \propto _{{19}} \omega ^{2} }}{\tau } - \frac{{b^{2} \propto _{{19}} \propto _{9} \omega ^{2} }}{\tau } \\ & - \frac{{b^{2} \propto _{1} \propto _{{19}} \propto _{9} \omega ^{2} }}{\tau } - a_{3} b^{4} \propto _{{17}} \omega ^{3} - b^{2} \propto _{{18}} \propto _{9} \omega ^{3} - b^{2} \propto _{1} \propto _{{19}} \propto _{9} \omega ^{3} \\ & - \frac{{a_{3} b^{2} \propto _{{17}} \propto _{9} \omega ^{3} }}{\tau } - a_{3} b^{2} \propto _{{18}} \omega ^{4} - a_{3} b^{2} \propto _{{17}} \propto _{9} \omega ^{4} - \frac{{ \propto _{3} \propto _{{18}} \propto _{9} \omega ^{4} }}{\tau } - a_{3} \propto _{{18}} \propto _{9} \omega ^{5} , \\ k_{{11}} = & \frac{{\beta _{{22}} }}{{\beta _{{11}} }},k_{{22}} = \frac{{\beta _{{33}} }}{{\beta _{{11}} }},k_{{33}} = \frac{{\beta _{{44}} }}{{\beta _{{11}} }},\gamma _{{11}} = 1 - \propto _{6} ,\gamma _{{22}} = - 2b^{2} + b^{2} \propto _{6} - \propto _{2} \propto _{7} - \propto _{8} \omega ^{2} + \propto _{6} \propto _{8} \omega , \\ \gamma _{{33}} = & b^{4} - b^{2} \propto _{2} \propto _{7} + b^{2} \propto _{8} \omega ^{2} ,k_{{44}} = \frac{{\gamma _{{22}} }}{{\gamma _{{11}} }},k_{{55}} = \frac{{\gamma _{{33}} }}{{\gamma _{{11}} }}. \\ \end{aligned}$$

On solving (40–44), a sixth and fourth order homogeneous differential equations is obtained as given by:

45 $$\left( {D^{6} - k_{11} D^{4} + k_{22} D^{2} - k_{33} } \right)\left( {\phi^{*} (x),\,\theta^{*} (x)N^{*} (x)} \right) = 0,$$

46 $$\left( {D^{4} - k_{44} D^{2} - k_{55} } \right)(\varphi_{2}^{*} (x),\,\psi^{*} (x)) = 0.$$

The characteristic equation corresponding to Eqs. (45) and (46) respectively are:

47 $$\lambda^{6} - k_{11} \lambda^{4} + k_{22} \lambda^{2} - k_{33} = 0,$$

48 $$\delta^{4} - k_{44} \delta^{2} - k_{55} = 0.$$

The definitions for the coefficients $k_{11} ,\,k_{22} ,\,\,k_{33} ,\,\,k_{44} ,\,k_{55}$ involved are detailed in Appendix I.

The solution of Eq. (45) and (46) is expressed as:

49 $$\theta^{*} \left( x \right) = A_{1} e^{ - \lambda x} + A_{2} e^{{ - \lambda_{2} x}} + A_{3} e^{{ - \lambda_{3} x}} ,$$

50 $$N^{*} \left( x \right) = A_{1} L_{11} e^{{ - \lambda_{1} x}} + A_{2} L_{12} e^{{ - \lambda_{2} x}} + A_{3} L_{13} e^{{ - \lambda_{3} x}} ,$$

51 $$\varphi^{*} (x) = A_{1} S_{11} e^{{ - \lambda_{1} x}} + A_{2} S_{12} e^{{ - \lambda_{2} x}} + A_{3} S_{13} e^{{ - \lambda_{3} x}} ,$$

52 $$\varphi_{2}^{*} (x) = B_{1} e^{{( - \delta_{1} x)}} + B_{2} e^{{( - \delta_{2} x)}} ,$$

53 $$\psi^{*} \left( x \right) = B_{1} V_{11} e^{{ - \delta_{1} x}} + B_{2} V_{12} e^{{ - \delta_{2} x}} .$$

where

$$\begin{gathered} L_{11} = \frac{{ \propto_{10} }}{{\tau \left( {\delta_{14} - \lambda_{1}^{2} } \right)}} ,L_{12} = \frac{{ \propto_{10} }}{{\tau \left( {\delta_{14} - \lambda_{2}^{2} } \right)}} ,L_{13} = \frac{{ \propto_{10} }}{{\tau \left( {\delta_{14} - \lambda_{3}^{2} } \right)}} ,S_{11} = \frac{{\left( {\delta_{13} - \alpha_{17} \omega \lambda_{1}^{2} } \right)}}{{\left( {\delta_{15} - \delta_{19} \lambda_{1}^{2} } \right)}} , \hfill \\ S_{12} = \frac{{\left( {\delta_{13} - \alpha_{17} \omega \lambda_{2}^{2} } \right)}}{{\left( {\delta_{15} - \delta_{19} \lambda_{2}^{2} } \right)}} ,S_{13} = \frac{{\left( {\delta_{13} - \alpha_{17} \omega \lambda_{3}^{2} } \right)}}{{\left( {\delta_{15} - \delta_{19} \lambda_{3}^{2} } \right)}} ,V_{11} = \frac{{\alpha_{2} }}{{b^{2} - \delta_{12} \delta_{1}^{2} }} , V_{12} = \frac{{\alpha_{2} }}{{b^{2} - \delta_{12} \delta_{2}^{2} }}, \hfill \\ \end{gathered}$$

, $\lambda_{i} (i = 1,2,3)$ are roots of auxiliary Eq. (34) and $\delta_{p} (p = 1,\,\,2)$ are the roots of auxiliary Eq. (35) are listed in Appendix II.

Substituting from Eqs. (51) and (53) into Eq. (33), we get

54 $$u = \left( {\mathop \sum \limits_{p = 1}^{3} - S_{1p} \lambda_{p} A_{p} e^{{ - \lambda_{p} x}} - i b\mathop \sum \limits_{p = 1}^{2} B_{p} V_{1p} e^{{ - \delta_{p} x}} } \right){\text{ e}}^{{{\text{t}} + {\text{ibz}},{ }}}$$

55 $$w = ( i b \mathop \sum \limits_{p = 1}^{3} S_{1p} A_{p} e^{{ - \lambda_{p} x}} - \mathop \sum \limits_{p = 1}^{2} B_{p} V_{1p} \delta_{p} e^{{ - \delta_{p} x}} ){\text{ e}}^{{\omega {\text{t}} + {\text{ibz }}}} .$$

Substituting Eqs. (49), (50), (52) (54) and (55) into Eqs. (26–31), we obtain:

56 $$\sigma_{xx} = \left( {\mathop \sum \limits_{p = 1}^{3} A_{p} e^{{ - \lambda_{p} x}} \left( {S_{1p} \lambda_{p}^{2} - b^{2} \alpha_{11} S_{1p} - 1 - L_{1p} \propto_{12} } \right) + \mathop \sum \limits_{p = 1}^{2} B_{p} i b V_{1p} \delta_{p} e^{{ - \delta_{p} x}} \left( {1 - \alpha_{11} } \right)} \right){\text{ e}}^{{\omega {\text{t}} + {\text{ibz }}}} ,$$

57 $$\sigma_{zz} = \left( {\mathop \sum \limits_{p = 1}^{3} A_{p} e^{{ - \lambda_{p} x}} \left( {\alpha_{11} S_{1p} \lambda_{p}^{2} - b^{2} S_{1p} - 1 - L_{1p} \propto_{12} } \right) + \mathop \sum \limits_{p = 1}^{2} B_{p} i b V_{1p} \delta_{p} e^{{ - \delta_{p} x}} \left( {1 + \alpha_{11} } \right)} \right) e^{\omega t + ibz }$$

58 $$\sigma_{zx} = \left( {\mathop \sum \limits_{p = 1}^{3} A_{p} e^{{ - \lambda_{p} x}} \lambda_{p} S_{1p} ib \left( { - \alpha_{14} - \alpha_{13} } \right) + \mathop \sum \limits_{p = 1}^{2} B_{p} e^{{ - \delta_{p} x}} \left( {\alpha_{14} b^{2} V_{1p} + \alpha_{13} \delta_{p}^{2} V_{1p} - \alpha_{15} } \right)} \right){\text{ e}}^{{\omega {\text{t}} + {\text{ibz }}}}$$

59 $$M_{zy} = ( \propto_{16} \mathop \sum \limits_{p = 1}^{3} B_{p } e^{{ - \delta_{p} x}} )ib{\text{ e}}^{{\omega {\text{t}} + {\text{ibz }},{ }}}$$

60 $$M_{xy} = \left( { - \propto_{16} \mathop \sum \limits_{p = 1}^{3} B_{p } \delta_{p} e^{{ - \delta_{p} x}} } \right){\text{ e}}^{{\omega {\text{t}} + {\text{ibz }}}} .$$

Using Eqs. (49), (50), (56), (58) and (60) in Eqs. (17–19) for $x = 0$, we obtain

61 $$A_{1} \lambda_{1} + A_{2} \lambda_{2} + A_{3} \lambda_{3} = q_{0} \frac{{t^{2} e^{{ - \frac{t}{{t_{p} }}}} }}{{16 k t_{p}^{2} }},$$

62 $$A_{1} \left( { - L_{11} \lambda_{1} - a_{19} L_{11} } \right) - A_{2} \left( { - L_{12} \lambda_{2} - a_{19} L_{12} } \right) - A_{3} \left( { - L_{13} \lambda_{3} - a_{19} L_{13} } \right) = 0,$$

63 $$\begin{aligned} & A_{1} \left( {S_{11} \lambda_{1}^{2} - b^{2} \propto_{11} S_{11} - L_{11} \propto_{12} - 1} \right) + A_{2} \left( {S_{12} \lambda_{2}^{2} - b^{2} \propto_{11} S_{12} - L_{12} \propto_{12} - 1} \right) \\ & \,\,\,\, + A_{3} \left( {S_{13} \lambda_{3}^{2} - b^{2} \propto_{11} S_{13} - L_{13} \propto_{12} - 1} \right) + B_{1} \left( {ibV_{11} \delta_{1} - ib\alpha_{11} \delta_{1} V_{11} } \right) \\ & \,\,\,\, + B_{2} \left( {ibV_{12} \delta_{2} - ib\alpha_{11} \delta_{2} V_{12} } \right) = 0, \\ \end{aligned}$$

64 $$\begin{aligned} & A_{1} \left( { - \lambda_{1} S_{11} \propto_{13} ib - \propto_{14} ibS_{11} \lambda_{1} } \right) + A_{2} \left( { - \lambda_{2} S_{12} \propto_{13} ib - \propto_{14} ibS_{12} \lambda_{2} } \right) \\ & \,\,\,\, + A_{3} \left( { - \lambda_{3} S_{13} \propto_{13} ib - \propto_{14} ibS_{13} \lambda_{3} } \right) + B_{1} \left( { \propto_{13} b^{2} V_{11} + \propto_{14} \delta_{1}^{2} V_{11} + \alpha_{15} } \right) \\ & \,\,\,\, + B_{1} \left( { \propto_{13} b^{2} V_{11} + \propto_{14} \delta_{1}^{2} V_{11} + \alpha_{15} } \right) = 0, \\ \end{aligned}$$

65 $$B_{1} \left( { - \propto_{16} \delta_{1} } \right) + B_{2} \left( { - \propto_{16} \delta_{2} } \right) = 0.$$

Since there is one non-homogeneous equation in Eq. (50) that may be used to calculate the constants $$A_{1}$$, $$A_{2}$$$$A_{3}$$, $$B_{1}$$ and $$B_{2} ,$$ Cramer’s method is applied.

66 $$A_{1} = \frac{{\Delta A_{1} }}{\Delta },\,A_{2} = \frac{{\Delta A_{2} }}{\Delta },\,A_{3} = \frac{{\Delta A_{3} }}{\Delta },\,B_{1} = \frac{{\Delta B_{1} }}{\Delta },\,B_{2} = \frac{{\Delta B_{2} }}{\Delta }$$

where ∆, $\Delta A_{1}$, $\Delta A_{2}$, $\Delta A_{3}$, $\Delta B_{1}$ and $\Delta B_{2}$ are listed in Appendix III.

## Particular case (neglecting electromagnetic field effect)

Neglecting electromagnetic field in the medium, the above problem reduces to a two-dimensional problem micropolar generalized thermoelastic medium with photothermal effect. By putting electromagnetic constants $\mu_{e} = 0,\,\,\varepsilon_{0} = 0$ in the field equations and constitutive relations and by incorporating this modification, the expressions of physical quantities can be deduced by adopting a similar approach, we get the thermo-elastic interactions in micropolar generalized thermoelasticity theory in the framework of photothermal theory is obtained and the results agree with Raddadi et al.^19^.

## Discussions on results

In this section, we delve into the intriguing aspects of wave propagation in a thermoelastic medium. Our approach involves numerical simulations to analyze crucial fields, including displacement components, temperature, stress components, couple stress, microrotation, and carrier density. Furthermore, Mathematica software has been used in order to evaluate the numerical values of the field quantities. For numerical computations, the material properties of Silicon as in^15^ are taken under consideration, which are as follows:

$$\begin{aligned} & \lambda = 3.64 \times 10^{10} \,\,{\text{Nm}}^{ - 2} ,\,\,\,\,\,\,\,\,\,\mu = 5.46 \times 10^{10} \,\,{\text{Nm}}^{ - 2} ,\,\,\,\,\,\,k = 10^{10} \,\,{\text{Nm}}^{ - 1} ,\,\,\,\,\,\,\,\,\rho = 2330\,\,{\text{gm}}^{ - 3} , \\ & \gamma = 0.779 \times 10^{ - 9} \,{\text{N}},\,\,\,\,\,\,j = 0.2 \times 10^{ - 19} \,{\text{m}}^{2} ,\,\,\,\,\,\,K^{*} = 150\,{\text{Wm}}^{ - 1} {\text{K}}^{ - 1} ,\,\,\,\,\,\,\,K^{*} = 3\,{\text{Wm}}^{ - 1} {\text{K}}^{ - 1} ,\,\,\,\, \\ & c_{e} = 650\,\,{\text{Jkg}}^{ - 1} \,{\text{K}}^{ - 1} ,\,\,\,\,\,\,T_{0} = 800\,\,{\text{K}},\,\,\,\,\tau = 5 \times 10^{ - 5} \,{\text{s}},\,\,\,\,\,E_{g} = 1.11\,{\text{eV}},\,\,\,\,D_{e} = 2.5 \times 10^{ - 3} \,\,{\text{m}}^{2} {\text{s}}^{ - 1} , \\ & d_{n} = - 9 \times 10^{ - 31} \,\,{\text{m}}^{3} ,\,\,\,\,\,s_{0} = 2\,{\text{ms}}^{ - 1} ,\,\,\,\,\,\,\,n_{0} = 10^{20} \,{\text{m}}^{ - 3} ,\,\,\,\,\,\,\alpha_{t} = 3 \times 10^{ - 6} \,{\text{K}}^{ - 1} ,\,\,\,\,\,a = 0.25,\,\,\,\,q_{0} = 10. \\ \end{aligned}$$

**Fig. 1:**
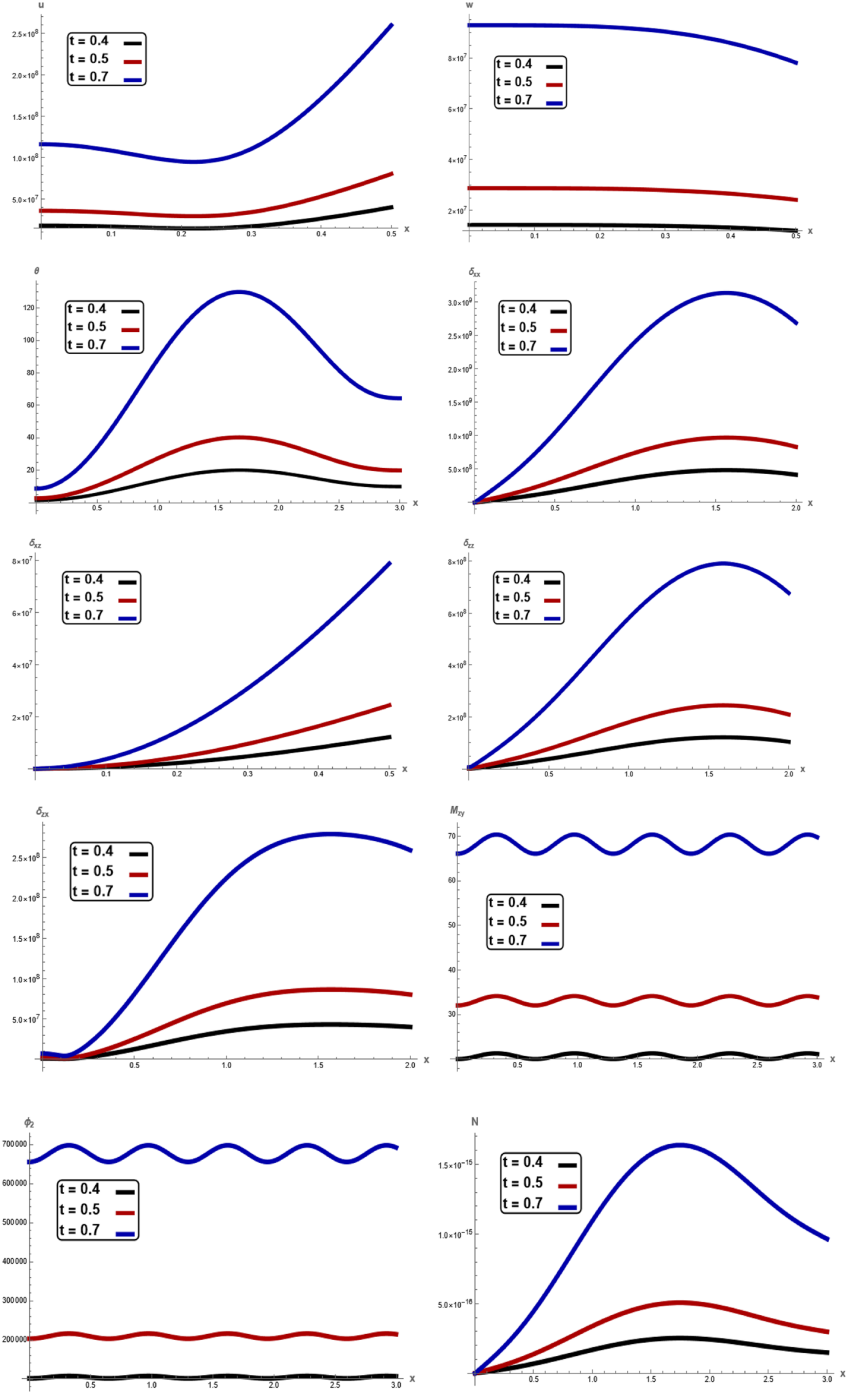
Variation of $u, \,w,\,\theta ,\,\sigma_{xx} ,\,\sigma_{zz} ,\, \sigma_{xz} ,{ }\sigma_{xz} \,, \,M_{zy} ,\,\varphi_{2}$ and $N$ with respect to $x{ - }axis$ for different values of time t.

**Fig. 2:**
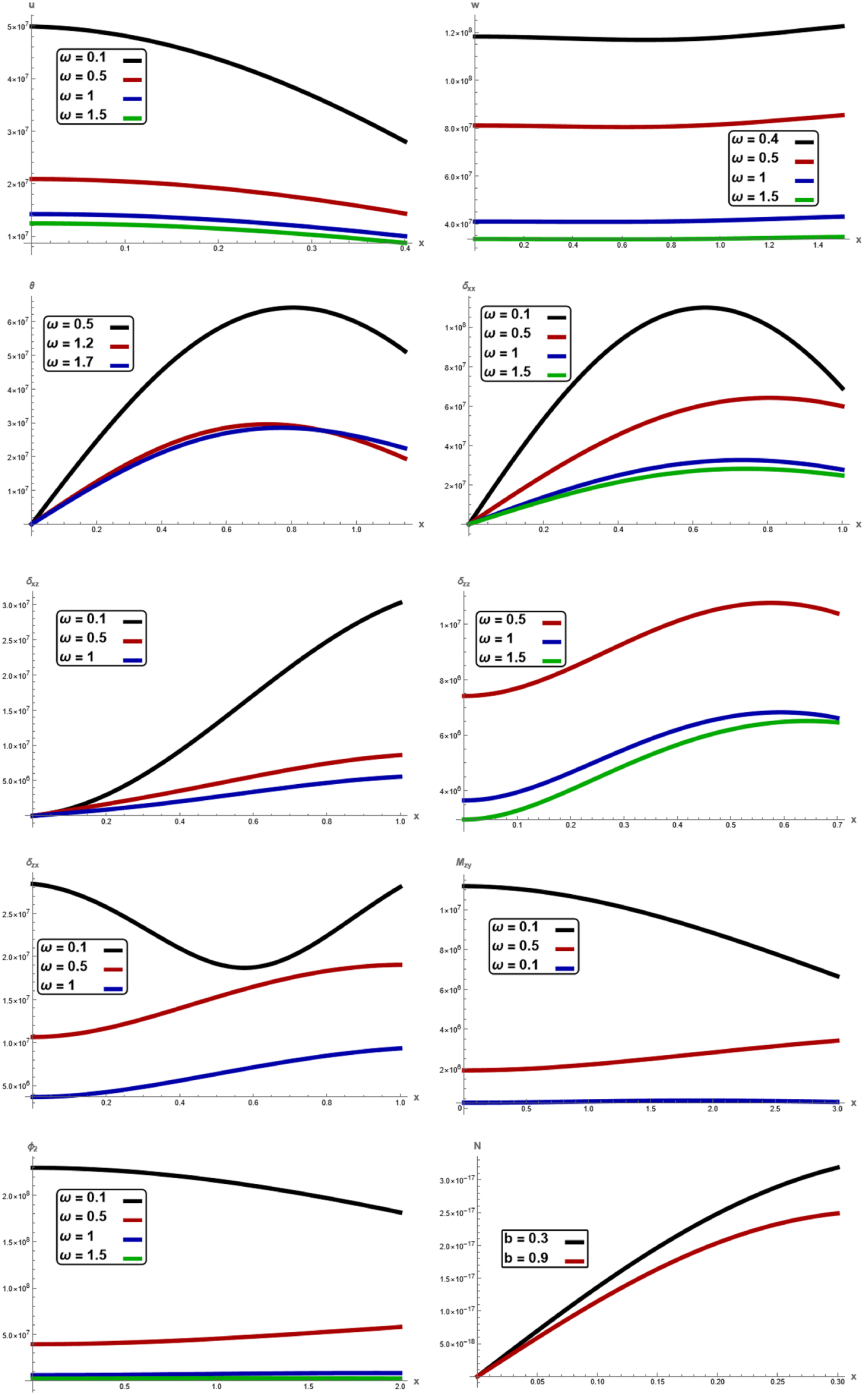
Variation of $u, \,w,\,\theta ,\,\sigma_{xx} ,\,\sigma_{zz} ,\, \sigma_{xz} ,{ }\sigma_{xz} \,, \,M_{zy} ,\,\varphi_{2}$ and $N$ with respect to $x{ - }axis$ for different values of angular frequency $\omega$.

**Fig. 3:**
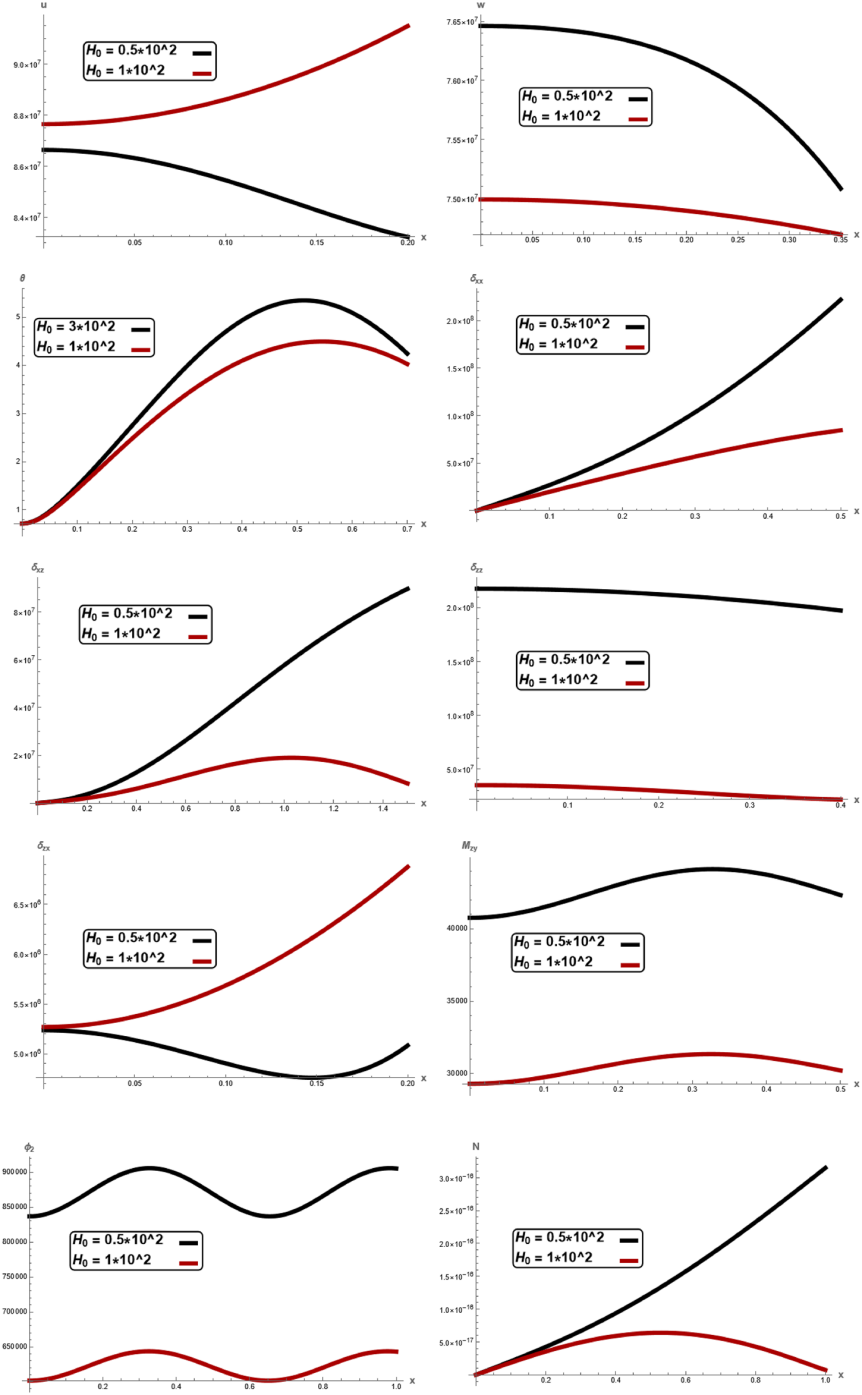
Variation of $u, \,w,\,\theta ,\,\sigma_{xx} ,\,\sigma_{zz} ,\, \sigma_{xz} ,{ }\sigma_{xz} \,, \,M_{zy} ,\,\varphi_{2}$ and $N$ with respect to $x{ - }axis$ for different values of magnetic field $H_{0}$.

**Fig.4:**
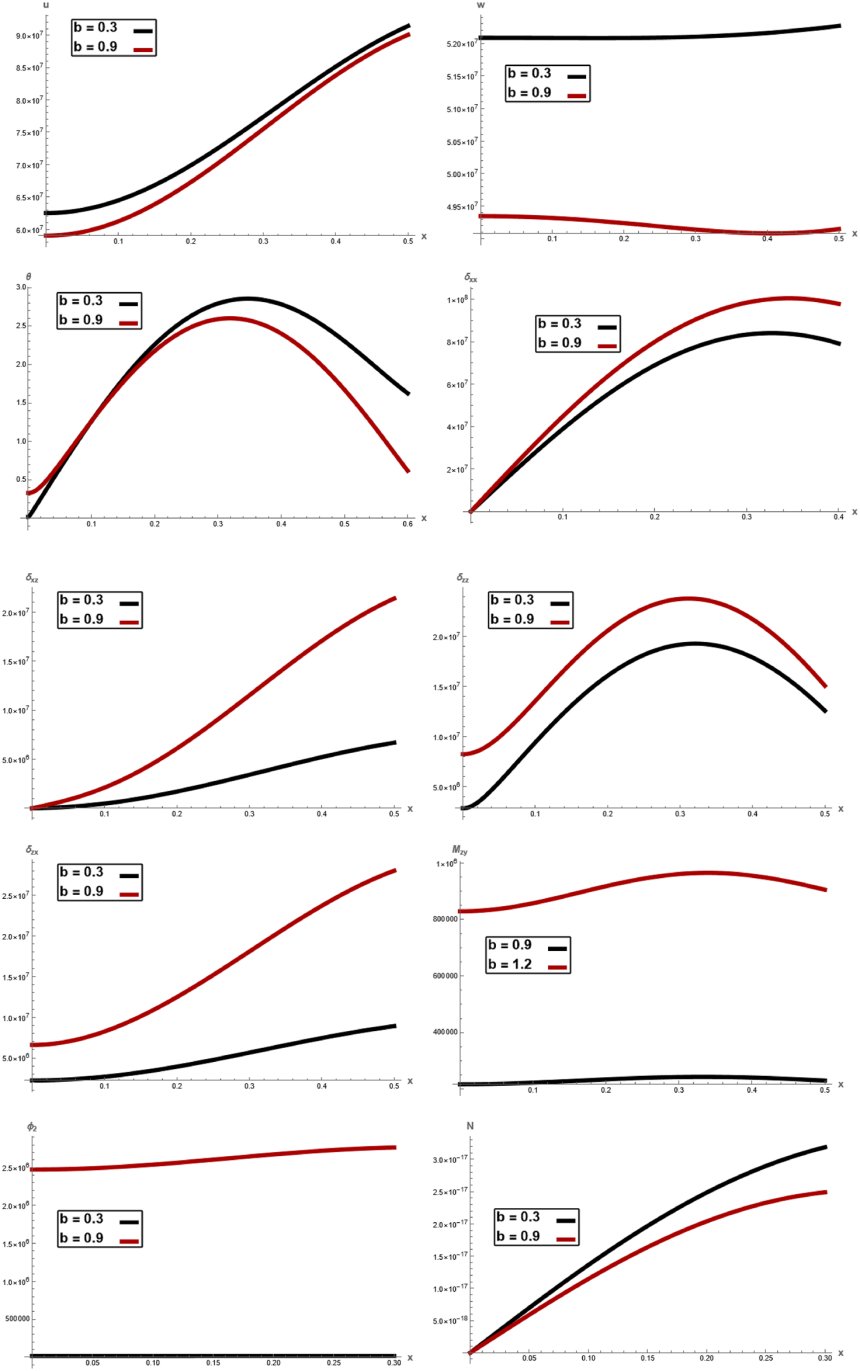
Variation of $u, \,w,\,\theta ,\,\sigma_{xx} ,\,\sigma_{zz} ,\, \sigma_{xz} ,{ }\sigma_{xz} \,, \,M_{zy} ,\,\varphi_{2}$ and $N$ with respect to $x{ - }axis$ for different values of laser pulse time *b*.

**Fig. 5:**
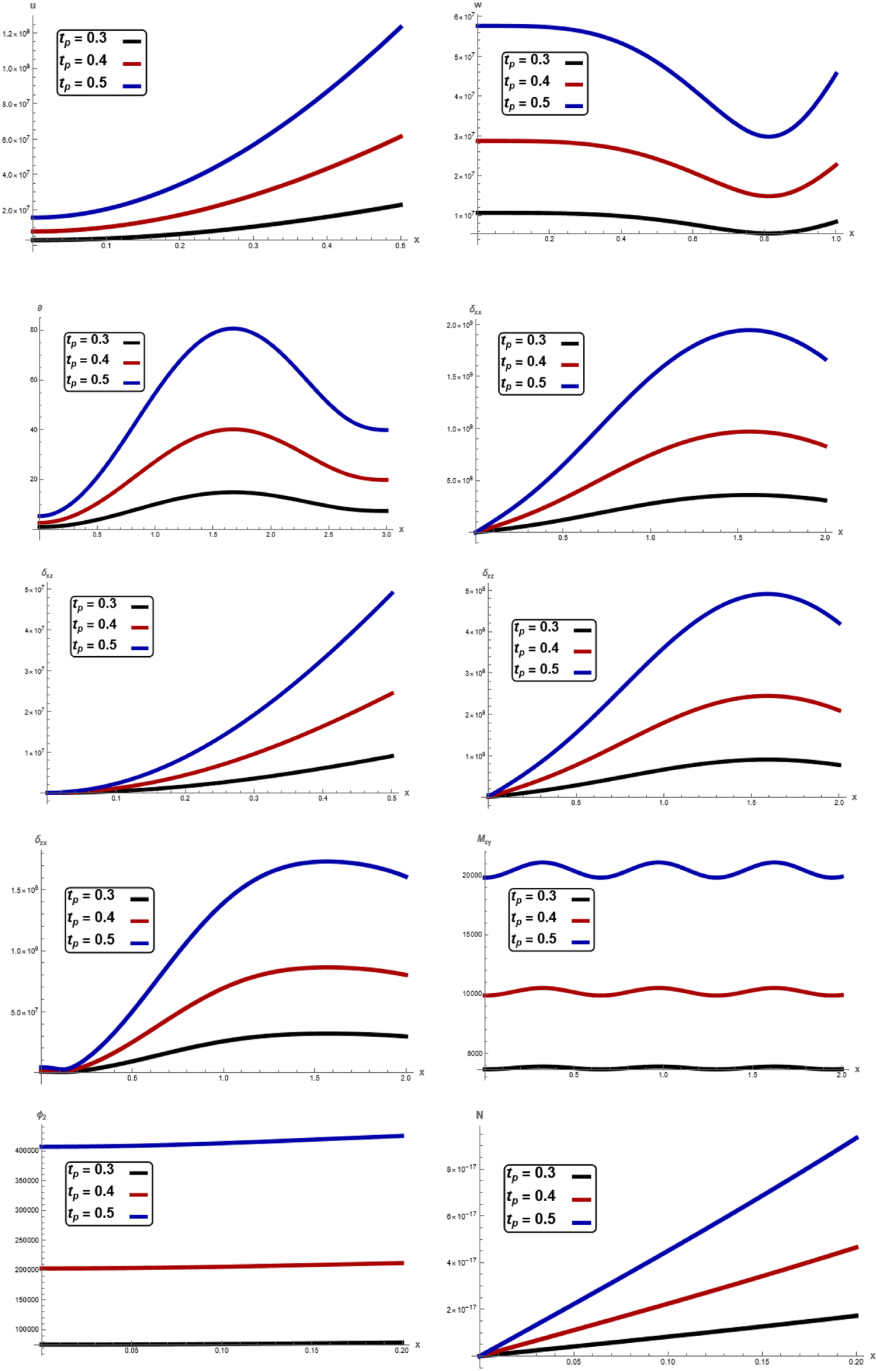
Variation of $u, \,w,\,\theta ,\,\sigma_{xx} ,\,\sigma_{zz} ,\, \sigma_{xz} ,{ }\sigma_{xz} \,, \,M_{zy} ,\,\varphi_{2}$ and $N$ with respect to $x{ - }axis$ for different values of laser pulse time $t_{p}$.

**Fig. 6:**
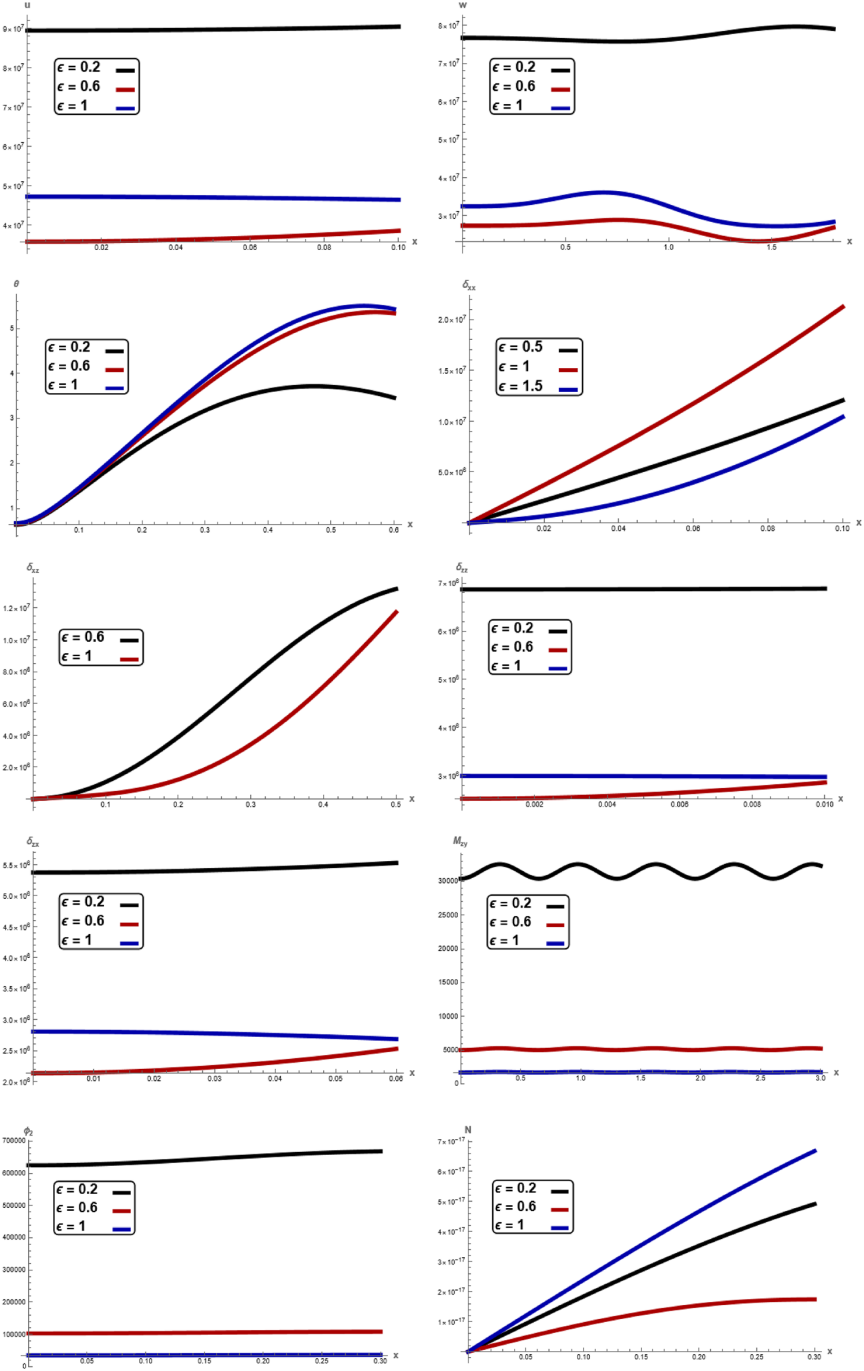
Variation of $u, \,w,\,\theta ,\,\sigma_{xx} ,\,\sigma_{zz} ,\, \sigma_{xz} ,{ }\sigma_{xz} \,, \,M_{zy} ,\,\varphi_{2}$ and $N$ with respect to $x{ - }axis$ for different values of electric permittivity $\varepsilon$.

**Fig. 7:**
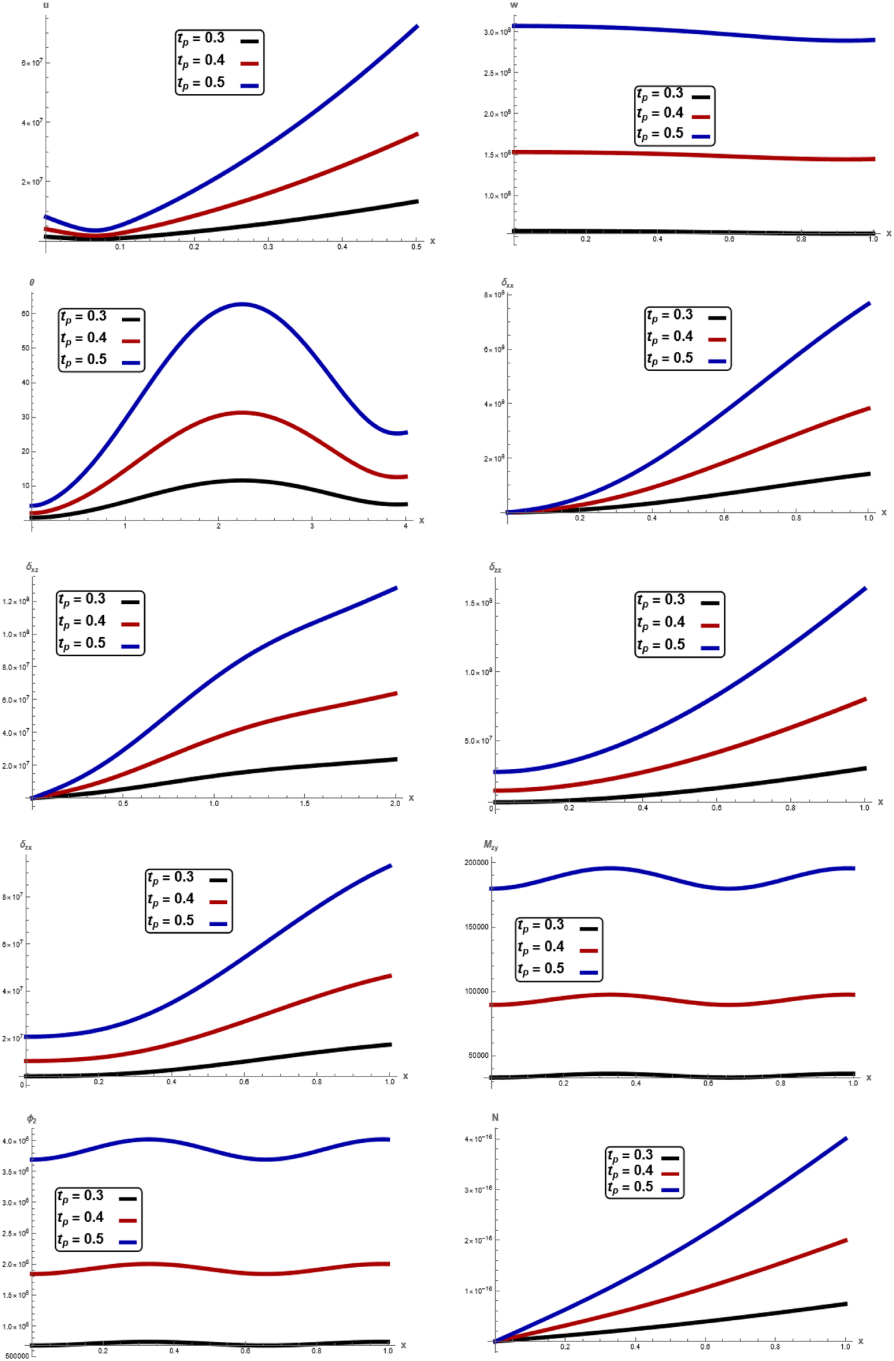
Variation of $u, \,w,\,\theta ,\,\sigma_{xx} ,\,\sigma_{zz} ,\, \sigma_{xz} ,{ }\sigma_{xz} \,, \,M_{zy} ,\,\varphi_{2}$ and $N$ with respect to $x{ - }axis$ for different values of laser pulse time $t_{p}$ without electromagnetic field.

The variations of all the quantities are shown in Figs. 1, 2, 3, 4, 5, 6, 7 to show the effect of different time $t,$ angular frequency $\omega ,\,$ magnetic field $H_{0} ,$ wave number $b,$ laser pulse time $t_{p} ,$ and electric permittivity $\varepsilon_{0} .$

In Fig. 1, clear difference in displacement components $u,\,w,$, temperature $\theta ,$ stress components $\sigma_{xx} ,\,\sigma_{zz} ,\,\sigma_{xz} ,$ couple stresses $M_{zy} ,$ $\sigma_{zx}$, microrotation $\varphi_{2}$ and carrier density $N$ with respect to $x -$ axis for different values time $t.$ The magnitude of $u,\,w,$ $\theta ,$ $\sigma_{xx} ,\,\sigma_{zz} ,\,\sigma_{xz} ,$ $M_{zy} ,$ $\varphi_{2}$ and $N$ notably increase with increasing of the time. In every instance of time, In every values of electric permittivity, all the curves in each figure have coincident initial coordinate $(0,\,0).$ These facts agree well with the boundary conditions. The results reveal that material stiffness strongly influences displacement, stress component, couple stress, microrotation and carrier density. It is noticed that the due to the time effect, the elastic waves (described by temperature $\theta$) on the surface are generated with a positive amplitude, which starts increasing when moving away from the source. After that, the elastic waves start showing periodic nature. In addition to this category depicts how the time has a significant impact on the physical quantities. This result is in a good agreement with the results obtained by Raddadi et al.^22^.

Figure 2, show the variations of the displacement components $$u,\,w,$$ temperature $$\theta ,$$ stress component $$\sigma_{xx} ,\,\sigma_{zz} ,\,\sigma_{xz} ,$$ couple stress $$\sigma_{zx} ,$$$$M_{zy}$$ and microrotation $$\varphi_{2}$$ with respect to $$x -$$ axis for different values angular frequency $$\omega .$$ It is observed that the magnitude of $$u,\,w,$$ $$\theta ,$$ $$\sigma_{xx} ,\,\sigma_{zz} ,\,\sigma_{xz} ,$$ $$M_{zy} ,$$ and $$\varphi_{2}$$ notably decrease with increasing of angular frequency, while $$\theta ,$$ increases with increasing of $$\omega .$$ In every value of angular frequency, the physical quantities satisfied the boundary conditions. The results reveal that material stiffness strongly influences displacement, stress component, couple stress, microrotation and carrier density. It is clearly observed that the mechanical waves are highly sensitive towards the characteristic angular frequency $$\omega$$. When the angular frequency increases, the values of physical quantities decrease. In the end, there is no discernible difference between the three curves. This result is in a good agreement with the results obtained by Jatain et al.^15^.

In Fig. 3, clear difference in displacement components $$u,\,w,$$, temperature $$\theta ,$$ stress component $$\sigma_{xx} ,\,\sigma_{zz} ,\,\sigma_{xz} ,$$ couple stress $$\sigma_{zx} ,$$$$M_{zy} ,$$ microrotation $$\varphi_{2}$$ and carrier density $$N$$ with respect to $$x -$$ axis for different values of magnetic field $$H_{0} .$$ It is noticed that the magnitude of $$w,$$ $$\theta ,$$ $$\sigma_{xx} ,\,\sigma_{zz} ,\,\sigma_{xz} ,$$ $$M_{zy} ,$$ $$\varphi_{2}$$ and $$N$$ notably decrease with increasing of magnetic field $$H_{0}$$, while $$u,\,\,\sigma_{zx}$$ increase with increasing of magnetic field. In every values of magnetic field, In every values of electric permittivity, all the curves in each figure have coincident initial coordinate $$(0,\,0).$$ These facts agree well with the boundary conditions. The results reveal that material stiffness strongly influences displacement, stress component, couple stress, microrotation and carrier density. When comparing the magnitude of the physical quantities for three different values of $$H_{0}$$, we found the fact that the effect of a magnetic field corresponds to the term signifying positive forces that tend to accelerate the metal particles. This result is in a good agreement with the results obtained by Raddadi et al.^24^.

Figure 4, shows the variations of the displacement components $$u,\,w,$$ temperature $$\theta ,$$ stress component $$\sigma_{xx} ,\,\sigma_{zz} ,\,\sigma_{xz} ,$$ couple stress $$\sigma_{zx} ,$$$$M_{zy} ,$$ microrotation $$\varphi_{2}$$ and $$N$$ with respect to $$x -$$ axis for different values wave number $$b.$$ It is observed that the magnitude of $$u,\,w,$$ $$\theta ,$$ and $$N$$ decrease with increasing of wave number $$b$$, while the magnitude of $$\sigma_{xx} ,\,\sigma_{zz} ,\,\sigma_{xz} ,$$$$\sigma_{zx} ,$$$$M_{zy} ,$$ and $$\varphi_{2}$$ increase with increasing of $$b,$$ as well, $$\sigma_{xz} ,\,\sigma_{zx} ,$$$$u$$ and $$N$$ increase with increasing of axial $$x.$$ In every value of wave number, In every values of electric permittivity, all the curves in each figure have coincident initial coordinate $$(0,\,0).$$ These facts agree well with the boundary conditions. This type of analysis helps identify regions where the system exhibits high responsiveness, as well as areas where it remains relatively unaffected by parameter changes. It is also observed that as value of $$x$$ increases, the magnitude of $$u,\,N,\,\sigma_{xz} ,\,\sigma_{xx}$$ increase rapidly and beyond a certain point in the region which agrees with the Green- Naghdi theory of thermoelasticity. The one common observation in physical quantities is higher the value of $$b$$, higher is the amplitude. This result is in a good agreement with the results obtained by Jatain et al.^15^.

In Fig. 5, clear difference in displacement components $$u,\,w,$$, temperature $$\theta ,$$ stress component $$\sigma_{xx} ,\,\sigma_{zz} ,\,\sigma_{xz} ,$$ couple stress $$\sigma_{zx} ,$$$$M_{zy} ,$$ microrotation $$\varphi_{2}$$ and carrier density $$N$$ with respect to of $$x -$$ axis for different values of laser pulse time $$t_{p} .$$ It is observed that the magnitude of $$u,$$ $$w,$$ $$\theta ,$$ $$\sigma_{xx} ,\,\sigma_{zz} ,\,\sigma_{xz} ,\sigma_{zx}$$ $$M_{zy}$$ and $$N$$ notably increase with increasing of laser pulse time, while $$\varphi_{2}$$ increases and decreases with increasing of laser pulse time. In every values of laser pulse time, the physical quantities satisfied the boundary conditions. Due to the photothermal effect, the elastic waves on the surface are generated with positive amplitude, which starts increasing when moving away from the source. After that, the elastic waves start showing periodic nature. Both the physical quantities viz. $$\sigma_{xx}$$ and $$\sigma_{zx}$$ show similar sensitivity towards $$t_{p}$$. Starting from a positive value, then showing an oscillatory nature with increasing amplitude as axial $$x$$ keeps increasing. Thus, it can be said that for higher values of $$x$$, we can find the same values of $$t{}_{p}$$, which can keep the amplitude in a controlled range. Varying $$t{}_{p}$$ in a system can significantly influence its behavior and performance, making sensitivity analysis a crucial tool for understanding the underlying dynamics. These results obey the physical properties of photo-thermoelasticity theory. This result is in a good agreement with the results obtained by Jatain et al.^15^.

Figure 6, reveals variation of displacement components $$u,\,w,$$ temperature $$\theta ,$$ stress component $$\sigma_{xx} ,\,\sigma_{zz} ,\,\sigma_{xz} ,$$ couple stress $$\sigma_{zx} ,$$$$M_{zy} ,$$ microrotation $$\varphi_{2}$$ and carrier density $$N$$ with respect to of $$x -$$ axis for different values of electric permittivity $$\varepsilon_{0} .$$ It is noticed that the magnitude of $$u,$$ $$w,$$ $$\sigma_{zx} ,\,M_{zy}$$ $$M_{zy}$$ and $$\varphi_{2}$$ decrease with increasing of electric permittivity, while $$\theta$$ increases with increasing of electric permittivity. Furthermore $$\sigma_{xx} ,\,\,N$$ increase and decrease with increasing of electric permittivity. In every values of electric permittivity, all the curves in each figure have coincident initial coordinate $$(0,\,0).$$ These facts agree well with the boundary conditions. The results reveal that material stiffness strongly influences displacement, stress component, couple stress, microrotation and carrier density. Understanding the relationship between parameters and outputs is essential for optimization, control, and decision-making processes, as it provides insight into which electric permittivity most significantly impact the system’s behavior. Overall, the physical quantities reveals that the behavior is highly dependent on the effects of $$\varepsilon_{0}$$, with increasing $$\varepsilon_{0}$$ leading to increase in the amplitude and variations affecting the physical quantities at different distances. This pattern is crucial for understanding physical quantities in materials and systems with amplitude dependencies. Additionally, recognizing points of high sensitivity can guide the design of more robust systems that perform consistently across a range of operating conditions, which frequently aligns with^17^.

Figures 7 demonstrate the influence of characteristic time $$t_{p}$$ on the displacement components $$u,\,w,$$ temperature $$\theta ,$$ stress component $$\sigma_{xx} ,\,\sigma_{zz} ,\,\sigma_{xz} ,$$ couple stress $$\sigma_{zx} ,$$$$M_{zy} ,$$ microrotation $$\varphi_{2}$$ and carrier density $$N$$ with respect to distance $$x$$ for without electromagnetic field. It is clearly reveals that the physical quantities $$u,\,w,$$ $$\theta ,$$ $$\sigma_{xx} ,\,\sigma_{zz} ,\,\sigma_{xz} ,$$ $$\sigma_{zx} ,$$ $$M_{zy} ,$$ and $$N$$ is greatly influenced by $$t_{p} .$$ Due to the photothermal effect, the elastic waves on the surface are generated with a positive amplitude, which starts increasing when moving away from the source. After that, the elastic waves start showing periodic nature. It is clearly evident that the amplitude of the physical quantities is maximum for $$t_{p} =$$ 0.5, and it reduces when the value of $$t_{p}$$ is reduced, i.e., for $$t_{p} = 0.4$$ for $$t_{p} = 0.3$$. In other words, the physical quantities reduce when the value of $$t_{p}$$ is reduced in terms of amplitude. Overall, the figures demonstrate that the physical quantities is more sensitive to changes in $$t_{p}$$, with increasing $$t_{p}$$ significantly suppressing the physical quantities, which is crucial in applications related to photothermal materials and thermoelasticity, where directional dependencies play a key role in mechanical responses. This result is in a good agreement with the results obtained by Raddadi et al.^19^.

## Conclusion

The present study provides a mathematical model to investigate the photothermal effect on the behavior of stress components, couple stress, displacement components, temperature, microrotation and carrier density in a homogeneous micropolar electro-magneto-thermoelastic medium under the framework of GN theory (type III) using the method of normal mode analysis. Through the application of appropriate boundary conditions, several key findings emerge: All the physical quantities satisfy the boundary conditions and obtain nonzero values only in the bounded region of space which supports the notion of Green- Naghdi theory of electro-magneto-thermoelasticity.

- Numerical results and analysis show that there is a significant effect of time, angular frequency, wave number, magnetic field, electric field and laser pulse time on the distribution of various enhances the temperature, displacement components, stress components, couple stress, microrotation and carrier density.
- Temperature variation influence physical quantities amplitudes, highlighting the critical role of semiconductor dynamics at solid interfaces.
- It is further observed that the presence of photothermal effect also influences the magnitude of physical quantities under investigation, thus confirming that all physical quantities are very sensitive to the photothermal effect.
- The influence of micropolar, photothermal and the boundary conditions introduced play a significant role in the investigation of thermoelastic medium deformation.
- Major changes have been visualized between the plotted curves related to all physical quantities due to the presence electric permittivity and magnetic field parameter in a wide range of the distance which reflects that electric permittivity and magnetic field parameter dominates every physical quantities a very diminutive range of distance.
- Based on the graphical representation, it can be concluded that the laser pulse time is having a increasing effect on each physical quantities, while the temporal variable is directly proportional to the amplitude each physical quantities.
- The derivation, analysis, and results discussed in this paper can be applied to designers of new materials and research in materials science as well as to those working on the second acoustic effect.
- The methods used in the presence article are applicable to a wide range of problems in thermodynamics and thermoelasticity.

## Supplementary Information

Supplementary Information.

## Data Availability

The datasets used and/or analyzed during the current study available from the corresponding author on reasonable request.

## List of symbols

$$\rho$$: Material density ($${\text{kg}}\,{\text{m}}^{ - 3}$$)
$k,\,\alpha ,\,\beta ,\,\gamma$: Micropolar material constants (${\text{N}}\,{\text{m}}^{ - 2}$)
$$T_{0}$$: Uniform temperature ($$K$$)
$$c_{e}$$: Specific heat ($${\text{kg}}^{ - 1} {\text{K}}^{ - 1}$$)
$$F_{i}$$: Lorentz forces
$$\lambda ,\,\,\mu$$: Lame’s constants ($${\text{N}}\,{\text{m}}^{ - 2}$$)
$\beta_{1} = (3\lambda + 2\mu + k)\alpha_{t}$: The thermal expansion of volume
$\gamma_{n} = (3\lambda + 2\mu )d_{n}$: Deformation potential coefficient
$$j$$: Microinertia (m^2^)
$T$: Absolute thermodynamic temperature (K)
$K^{*}$: Material constants characteristics of the theory (${\text{Wm}}^{ - 1} {\text{K}}$)
$$\varepsilon_{0}$$: Electric permittivity ($${\text{F}}\,{\text{m}}^{ - 1}$$)
$\alpha_{t}$: Coefficient of linear thermal expansion (${\text{K}}^{ - 1}$)
$K^{*}$: Material constant characteristic of the theory (${\text{Wm}}^{ - 1} {\text{K}}$)
$$k$$: Thermal conductivity ($${\text{N}}\,{\text{m}}^{ - 1}$$)
$E_{g}$: Semiconducting energy gap (ev)
$\sigma_{ij}$: Components of stress tensor (${\text{N}}\,{\text{m}}^{ - 2}$)
$$m_{ij}$$: Couple stress ($${\text{N}}\,{\text{m}}^{ - 2}$$)
$d{}_{n}$: Represent electronic deformation coefficient (m^3^)
$$\mu_{e}$$: Magnetic permeability ($${\text{H}}\,{\text{m}}^{ - 1}$$)
$$N$$: Carrier density ($${\text{cm}}^{ - 3}$$)
$\tau$: Photo-generated carrier life time (s)
$$\delta_{ij}$$: Kronekr’s delta
$$t$$: Time (s)
$$b$$: Wave number ($${\text{cm}}\,{\text{s}}^{ - 1}$$)
$$\omega$$: Angular frequency ($${\text{rad}}\,{\text{s}}^{ - 1}$$)
$D_{e}$: Carrier diffusion coefficient (${\text{m}}^{2} {\text{s}}^{ - 1}$)
$u_{i}$: Components of displacement vector $\overrightarrow {{\text{u}}} \,({\text{m}})$
$n_{0}$: Electron number density (${\text{m}}^{ - 3}$)
$s_{0}$: Surface recombination speed (${\text{ms}}^{ - 1}$)
$\overrightarrow {E}$: Electric field vector (${\text{V}}\,{\text{m}}^{ - 1}$)
$\overrightarrow {H}$: Magnetic field vector (${\text{Col}}\,{\text{cm}}^{ - 1} \,{\text{s}}^{ - 1}$)
$$\varphi_{2}$$: Microrotation
