# Supplementary material for: Photothermal interactions in micropolar generalized thermoelastic medium subjected to electromagnetic field
Source: Sci Rep. 2025 Nov 12;15:39576. doi: 10.1038/s41598-025-23882-3 (PMC12612191; doi:10.1038/s41598-025-23882-3)
Supplement: Supplementary file 1 — Supplementary Information. [file 41598_2025_23882_MOESM1_ESM.docx]

**Appendix I**

$$K_{11}=$$

$$K_{22}=$$

$$K_{33}=$$

__

_where_

$\beta_{11}=\delta_{18}$_,_

$$\beta_{22}=-\delta_{13}-\delta_{11}\delta_{18}-\delta_{14}\delta_{18}-\propto_{1}\delta_{19},$$

$\beta_{33}=\delta_{11}\delta_{13}+\delta_{13}\delta_{14}+\propto_{1}\delta_{15}+\delta_{11}\delta_{14}\delta_{18}+\propto_{1}\delta_{14}\delta_{19}+\frac{a_{2}\propto_{10}\delta_{19}}{\tau}$_,_

$\beta_{44}=-\delta_{11}\delta_{13}\delta_{14}-\propto_{1}\delta_{14}\delta_{15}-\frac{a_{2}\propto_{10}\delta_{15}}{\tau}$_._

**Appendix II**

$$\lambda_{1}=\frac{1}{\sqrt{6}}\left( \sqrt{\left( 22^{1/3}K_{11}^{2}-62^{1/3} K_{22}+\left( \eta_{2}+6\sqrt{\eta_{1}} \right)^{2/3}+2K_{11}\left( \eta_{3}+3\left( 9K_{33}+\sqrt{\eta_{1}} \right) \right)^{1/3} \right)/{\left( \eta_{3}+3\left( 9K_{33}+\sqrt{\eta_{1}} \right) \right)^{1/3}}} \right)$$

$$\lambda_{2}=\frac{1}{2\sqrt{3}}\left( \sqrt{\left( \left( -1-ⅈ\sqrt{3} \right)\left( \eta_{2}+6\sqrt{\eta_{1}} \right)^{2/3}+4K_{11}\left( \eta_{3}+3\left( 9K_{33}+\sqrt{\eta_{1}} \right) \right)^{1/3}+{K_{11}}^{2}-2.519+4.364ⅈ+( 7\text{.5595}-13.0934ⅈ )K_{22} \right)/{\left( \eta_{3}+3\left( 9K_{33}+\sqrt{\eta_{1}} \right) \right)^{1/3}}} \right)$$

$$\lambda_{3}=\frac{1}{\sqrt{6}}(\surd({(-2{(-2)}^{1/3}{K_{11}}^{2}+6{(-2)}^{1/3}K_{22}+{(-2)}^{2/3}{(\eta_{3}+27K_{33}+3\sqrt{\eta_{1}})}^{2/3}+2K_{11}{(\eta_{3}+3(9K_{33}+\sqrt{\eta_{1}}))}^{1/3})}/{{(\eta_{3}+3(9K_{33}+\sqrt{\eta_{1}}))}^{1/3}}))$$

$$\delta_{1}=\sqrt{\frac{\eta_{4}}{2}} ; \delta_{2}=\sqrt{\frac{\eta_{5}}{2}}$$

where,$\eta_{1}=-3\left( {K_{11}}^{2}-4K_{22} \right){K_{22}}^{2}+6K_{11}\left( 2{K_{11}}^{2}-9K_{22} \right)K_{33}+81{K_{33}}^{2}; \eta_{2}=4{K_{11}}^{3}-18K_{11}K_{22}+54K_{33}; \eta_{4}=2{K_{11}}^{3}-9K_{11}K_{22}; \eta_{4}=K_{44}-\sqrt{{K_{44}}^{2}+4K_{55}}; \eta_{5}=K_{44}+\sqrt{{K_{44}}^{2}+4K_{55}}.$

Appendix III

$$\Delta=\alpha_{16}(b^{2}V_{11}(-1+\alpha_{11})(\alpha_{13}+\alpha_{14})\delta_{1}\delta_{2}((L_{13}S_{11}+{L_{11}S}_{12}-L_{13}S_{12}-L_{11}S_{13}+L_{12}(-S_{11}+S_{13}))\lambda_{1}\lambda_{2}\lambda_{3}+a_{19}(L_{13}(S_{11}-S_{12})\lambda_{1}\lambda_{2}+L_{12}(-S_{11}+S_{13})\lambda_{1}\lambda_{3}+L_{11}(S_{12}-S_{13})\lambda_{2}\lambda_{3}))-{b^{2}V}_{12}(-1+\alpha_{11})(\alpha_{13}+\alpha_{14})\delta_{1}\delta_{2}((L_{13}S_{11}+L_{11}S_{12}-L_{13}S_{12}-L_{11}S_{13}+L_{12}(-S_{11}+S_{13}))\lambda_{1}\lambda_{2}\lambda_{3}+a_{19}(L_{13}(S_{11}-S_{12})\lambda_{1}\lambda_{2}+L_{12}(-S_{11}+S_{13})\lambda_{1}\lambda_{3}+L_{11}(S_{12}-S_{13})\lambda_{2}\lambda_{3}))-(b^{2}V_{11}\alpha_{13}+\alpha15+V_{11}\alpha_{14}\delta_{1}^{2})\delta_{2}(-L_{12}(1+b^{2}S_{11}\alpha_{11}+L_{11}\alpha_{12}-S_{11}\lambda_{1}^{2})(a_{19}+\lambda_{2})\lambda_{3}+L_{11}(a_{19}+\lambda_{1})(1+b^{2}S_{12}\alpha_{11}+L_{12}\alpha_{12}-S_{12}\lambda_{2}^{2})\lambda_{3}+L_{13}(1+b^{2}S_{11}\alpha_{11}+L_{11}\alpha_{12}-S_{11}\lambda_{1}^{2})\lambda_{2}(a_{19}+\lambda_{3})-L_{13}\lambda_{1}(1+b^{2}S_{12}\alpha_{11}+L_{12}\alpha_{12}-S_{12}\lambda_{2}^{2})(a_{19}+\lambda_{3})-L_{11}(a_{19}+\lambda_{1})\lambda_{2}(1+b^{2}S_{13}\alpha_{11}+L_{13}\alpha_{12}-S_{13}\lambda_{3}^{2})+L_{12}\lambda_{1}(a_{19}+\lambda_{2})(1+b^{2}S_{13}\alpha_{11}+L_{13}\alpha_{12}-S_{13}\lambda_{3}^{2}))+\delta_{1}(b^{2}V_{12}\alpha_{13}+\alpha_{15}+V_{12}\alpha_{14}\delta_{2}^{2})(-L_{12}(1+b^{2}S_{11}\alpha_{11}+L_{11}\alpha_{12}-S_{11}\lambda_{1}^{2})(a_{19}+\lambda_{2})\lambda_{3}+L_{11}(a_{19}+\lambda_{1})(1+b^{2}S_{12}\alpha_{11}+L_{12}\alpha_{12}-S_{12}\lambda_{2}^{2})\lambda_{3}+L_{13}(1+b^{2}S_{11}\alpha_{11}+L_{11}\alpha_{12}-S_{11}\lambda_{1}^{2})\lambda_{2}(a_{19}+\lambda_{3})-L_{13}\lambda_{1}(1+b^{2}S_{12}\alpha_{11}+L_{12}\alpha_{12}-S_{12}\lambda_{2}^{2})(a_{19}+\lambda_{3})-L_{11}(a_{19}+\lambda_{1})\lambda_{2}(1+b^{2}S_{13}\alpha_{11}+L_{13}\alpha_{12}-S_{13}\lambda_{3}^{2})+L_{12}\lambda_{1}(a_{19}+\lambda_{2})(1+b^{2}S_{13}\alpha_{11}+L_{13}\alpha_{12}-S_{13}\lambda_{3}^{2})))$$

$$A_{1}=(ⅇ^{-\frac{t}{t_{p}}} Kq_{0} t^{2} t_{p}^{2}(-L_{13}\delta_{2}(b^{2}S_{12}V_{11}(-1+\alpha_{11})(\alpha_{13}+\alpha_{14})\delta_{1}\lambda_{2}-(b^{2}V_{11}\alpha_{13}+\alpha_{15}+V_{11}\alpha_{14}\delta_{1}^{2})(1+b^{2}S_{12}\alpha_{11}+L_{12}\alpha_{12}-S_{12}\lambda_{2}^{2}))(a_{19}+\lambda_{3})+L_{13}\delta_{1}(b^{2}S_{12}V_{12}(-1+\alpha_{11})(\alpha_{13}+\alpha_{14})\delta_{2}\lambda_{2}-(b^{2}V_{12}\alpha_{13}+\alpha_{15}+V_{12}\alpha_{14}\delta_{2}^{2})(1+b^{2}S_{12}\alpha_{11}+L_{12}\alpha_{12}-S_{12}\lambda_{2}^{2}))(a_{19}+\lambda_{3})+L_{12}\delta_{2}(a_{19}+\lambda_{2})(b^{2}S_{13}V_{11}(-1+\alpha_{11})(\alpha_{13}+\alpha_{14})\delta_{1}\lambda_{3}-(b^{2}V_{11}\alpha_{13}+\alpha_{15}+V_{11}\alpha_{14} \delta_{1}^{2})(1+b^{2}S_{13}\alpha_{11}+L_{13}\alpha_{12}-S_{13}\lambda_{3}^{2}))-L_{12}\delta_{1}(a_{19}+\lambda2)(b^{2}S_{13}V_{12}(-1+\alpha_{11})(\alpha_{13}+\alpha_{14})\delta_{2}\lambda_{3}-(b^{2}V_{12}\alpha_{13}+\alpha_{15}+V_{12}\alpha_{14}\delta_{2}^{2})(1+b^{2}S_{13}\alpha_{11}+L_{13}\alpha_{12}-S_{13}\lambda_{3}^{2})))) /\Delta$$

$$A_{2}=-((ⅇ^{-\frac{t}{t_{p}}} Kq_{0} t^{2} t_{p}^{2}(-\delta_{2}(-((-((ⅈbV_{11}\delta_{1}-ⅈbV_{11}\alpha_{11}\delta_{1})(-ⅈbS_{11}\alpha_{13}\lambda_{1}-ⅈbS_{11}\alpha_{14}\lambda_{1}))+(b^{2}V_{11}\alpha_{13}+\alpha_{15}+V_{11}\alpha_{14}\delta_{1}^{2})(-1-b^{2}S_{11}\alpha_{11}-L_{11}\alpha_{12}+S_{11}\lambda_{1}^{2}))(-a_{19}L_{13}-L_{13}\lambda_{3}))+(-a_{19}L_{11}-L_{11}\lambda_{1})(-((ⅈbV_{11}\delta_{1}-ⅈbV_{11}\alpha_{11}\delta_{1})(-ⅈbS_{13}\alpha_{13}\lambda_{3}-ⅈbS_{13}\alpha_{14}\lambda_{3}))+(b^{2}V_{11}\alpha_{13}+\alpha_{15}+V_{11}\alpha_{14}\delta_{1}^{2})(-1-b^{2}S_{13}\alpha_{11}-L_{13}\alpha_{12}+S_{13}\lambda_{3}^{2})))+\delta_{1}(-((-((ⅈbV_{12}\delta_{2}-ⅈbV_{12}\alpha_{11}\delta_{2})(-ⅈbS_{11}\alpha_{13}\lambda_{1}-ⅈbS_{11}\alpha_{14}\lambda_{1}))+(b^{2}V_{12}\alpha_{13}+\alpha_{15}+V_{12}\alpha_{14}\delta_{2}^{2})(-1-b^{2}S_{11}\alpha_{11}-L_{11}\alpha_{12}+S_{11}\lambda_{1}^{2}))(-a_{19}L_{13}-L_{13}\lambda_{3}))+(-a_{19}L_{11}-L_{11}\lambda_{1})(-((ⅈbV_{12}\delta_{2}-ⅈbV_{12}\alpha_{11}\delta_{2})(-ⅈbS_{13}\alpha_{13}\lambda_{3}-ⅈbS_{13}\alpha_{14}\lambda_{3}))+(b^{2}V_{12}\alpha_{13}+\alpha_{15}+V_{12}\alpha_{14}\delta_{2}^{2})(-1-b^{2}S_{13}\alpha_{11}-L_{13}\alpha_{12}+S_{13}\lambda_{3}^{2})))))/\Delta$$

$$A_{3}=(ⅇ^{-\frac{t}{t_{p}}} Kq_{0} t^{2} t_{p}^{2}(-\delta_{2}(-((-((ⅈbV_{11}\delta_{1}-ⅈbV_{11}\alpha_{11}\delta_{1})(-ⅈbS_{11}\alpha_{13}\lambda_{1}-ⅈbS_{11}\alpha_{14}\lambda_{1}))+(b^{2}V_{11}\alpha_{13}+\alpha_{15}+V_{11}\alpha_{14}\delta_{1}^{2})(-1-b^{2}S_{11}\alpha_{11}-L_{11}\alpha_{12}+S_{11}\lambda_{1}^{2}))(-a_{19}L_{13}-L_{12}\lambda_{2}))+(-a_{19}L_{11}-L_{11}\lambda_{1})(-((ⅈbV_{11}\delta_{1}-ⅈbV_{11}\alpha_{11}\delta_{1})(-ⅈbS_{12}\alpha_{13}\lambda_{2}-ⅈbS_{12}\alpha_{14}\lambda_{2}))+(b^{2}V_{11}\alpha_{13}+\alpha_{15}+V_{11}\alpha_{14}\delta_{1}^{2})(-1-b^{2}S_{12}\alpha_{11}-L_{12}\alpha_{12}+S_{12}\lambda_{2}^{2})))+\delta_{1}(-((-((ⅈbV_{12}\delta_{2}-ⅈbV_{12}\alpha_{11}\delta_{2})(-ⅈbS_{11}\alpha_{13}\lambda_{1}-ⅈbS_{11}\alpha_{14}\lambda_{1}))+(b^{2}V_{12}\alpha_{13}+\alpha_{15}+V_{12}\alpha_{14}\delta_{2}^{2})(-1-b^{2}S_{11}\alpha_{11}-L_{11}\alpha_{12}+S_{11}\lambda_{1}^{2}))(-a_{19}L_{12}-L_{12}\lambda_{2}))+(-a_{19}L_{11}-L_{11}\lambda_{1}))(-((ⅈbV_{12}\delta_{2}-ⅈbV_{12}\alpha_{11}\delta_{2})(-ⅈbS_{12}\alpha_{13}\lambda_{2}-ⅈbS_{12}\alpha_{14}\lambda_{2}))+(b^{2}V_{12}\alpha_{13}+\alpha_{15}+V_{12}\alpha_{14}\delta_{2}^{2})(-1-b^{2}S_{12}\alpha_{11}-L_{12}\alpha_{12}+S_{12}\lambda_{2}^{2})))))/\Delta$$

$$B_{1}=(ⅇ^{-\frac{t}{t_{p}}} Kq_{0} t^{2} t_{p}^{2} \delta_{2}(((-1-b^{2}S_{11}\alpha_{11}-L_{11}\alpha_{12}+S_{11}\lambda_{1}^{2})(-ⅈbS_{12}\alpha_{13}\lambda_{2}-ⅈbS_{12}\alpha_{14}\lambda_{2})-(-ⅈbS_{11}\alpha_{13}\lambda_{1}-ⅈbS_{11}\alpha_{14}\lambda_{1})(-1-b^{2}S_{12}\alpha_{11}-L_{12}\alpha_{12}+S_{12}\lambda_{2}^{2}))(-a_{19}L_{13}-L_{13}\lambda_{3})-(-a_{19}L_{12}-L_{12}\lambda_{2})((-1-b^{2}S_{11}\alpha_{11}-L_{11}\alpha_{12}+S_{11}\lambda_{1}^{2})(-ⅈbS_{13}\alpha_{13}\lambda_{3}-ⅈbS_{13}\alpha_{14}\lambda_{3})-(-ⅈbS_{11}\alpha_{13}\lambda_{1}-ⅈbS_{11}\alpha_{14}\lambda_{1})(-1-b^{2}S_{13}\alpha_{11}-L_{13}\alpha_{12}+S_{13}\lambda_{3}^{2}))+(-a_{19}L_{11}-L_{11}\lambda_{1})((-1-b^{2}S_{12}\alpha_{11}-L_{12}\alpha_{12}+S_{12}\lambda_{2}^{2})(-ⅈbS_{13}\alpha_{13}\lambda_{3}-ⅈbS_{13}\alpha_{14}\lambda_{3})-(-ⅈbS_{12}\alpha_{13}\lambda_{2}-ⅈbS_{12}\alpha_{14}\lambda_{2})(-1-b^{2}S_{13}\alpha_{11}-L_{13}\alpha_{12}+S_{13}\lambda_{3}^{2}))))/\Delta$$

$$B_{2}=-((ⅇ^{-\frac{t}{t_{p}}} Kq_{0} t^{2} t_{p}^{2} \delta_{1}(((-1-b^{2}S_{11}\alpha_{11}-L_{11}\alpha_{12}+S_{11}\lambda_{1}^{2})(-ⅈbS_{12}\alpha_{13}\lambda_{2}-ⅈbS_{12}\alpha_{14}\lambda_{2})-(-ⅈbS_{11}\alpha_{13}\lambda_{1}-ⅈbS_{11}\alpha_{14}\lambda_{1})(-1-b^{2}S_{12}\alpha_{11}-L_{12}\alpha_{12}+S_{12}\lambda_{2}^{2}))(-a_{19}L_{13}-L_{13}\lambda_{3})-(-a_{19}L_{12}-L_{12}\lambda_{2})((-1-b^{2}S_{11}\alpha_{11}-L_{11}\alpha_{12}+S_{11}\lambda_{1}^{2})(-ⅈbS_{13}\alpha_{13}\lambda_{3}-ⅈbS_{13}\alpha_{14}\lambda_{3})-(-ⅈbS_{11}\alpha_{13}\lambda_{1}-ⅈbS_{11}\alpha_{14}\lambda_{1})(-1-b^{2}S_{13}\alpha_{11}-L_{13}\alpha_{12}+S_{13}\lambda_{3}^{2}))+(-a_{19}L_{11}-L_{11}\lambda_{1})((-1-b^{2}S_{12}\alpha_{11}-L_{12}\alpha_{12}+S_{12}\lambda_{2}^{2})(-ⅈbS_{13}\alpha_{13}\lambda_{3}-ⅈbS_{13}\alpha_{14}\lambda_{3})-(-ⅈbS_{12}\alpha_{13}\lambda_{2}-ⅈbS_{12}\alpha_{14}\lambda_{2})(-1-b^{2}S_{13}\alpha_{11}-L_{13}\alpha_{12}+S_{13}\lambda_{3}^{2}))))/\Delta$$
